# Effect of Rotigotine vs Placebo on Cognitive Functions Among Patients With Mild to Moderate Alzheimer Disease: A Randomized Clinical Trial

**DOI:** 10.1001/jamanetworkopen.2020.10372

**Published:** 2020-07-15

**Authors:** Giacomo Koch, Caterina Motta, Sonia Bonnì, Maria Concetta Pellicciari, Silvia Picazio, Elias Paolo Casula, Michele Maiella, Francesco Di Lorenzo, Viviana Ponzo, Clarissa Ferrari, Eugenia Scaricamazza, Carlo Caltagirone, Alessandro Martorana

**Affiliations:** 1 Department of Behavioral and Clinical Neurology, Santa Lucia Foundation Istituto di Ricerca e Cura a Carattere Scientifico (IRCCS), Rome, Italy; 2 Section of Human Physiology, University of Ferrara, Italy; 3 Unit of Statistics, IRCCS Istituto Centro San Giovanni di Dio Fatebenefratelli, Brescia, Italy; 4 Department of Systems Medicine, University of Rome Tor Vergata, Rome, Italy

## Abstract

**Importance:**

Impairment of dopaminergic transmission may contribute to cognitive dysfunction in Alzheimer disease (AD).

**Objective:**

To investigate whether therapy with dopaminergic agonists may affect cognitive functions in patients with AD.

**Design, Setting, and Participants:**

This phase 2, monocentric, randomized, double-blind, placebo-controlled trial was conducted in Italy. Patients with mild to moderate AD were enrolled between September 1, 2017, and December 31, 2018. Data were analyzed from July 1 to September 1, 2019.

**Interventions:**

A rotigotine 2 mg transdermal patch for 1 week followed by a 4 mg patch for 23 weeks (n = 47) or a placebo transdermal patch for 24 weeks (n = 47).

**Main Outcomes and Measures:**

The primary end point was change from baseline on the Alzheimer Disease Assessment Scale–Cognitive Subscale. Secondary end points were changes in Frontal Assessment Battery, Alzheimer Disease Cooperative Study–Activities of Daily Living, and Neuropsychiatric Inventory scores. Prefrontal cortex activity was evaluated by transcranial magnetic stimulation combined with electroencephalography.

**Results:**

Among 94 patients randomized (mean [SD] age, 73.9 [5.6] years; 58 [62%] women), 78 (83%) completed the study. Rotigotine, as compared with placebo, had no significant effect on the primary end point: estimated mean change in Alzheimer Disease Assessment Scale–Cognitive Subscale score was 2.92 (95% CI, 2.51-3.33) for the rotigotine group and 2.66 (95% CI, 2.31-3.01) for the placebo group. For the secondary outcomes, there were significant estimated mean changes between groups for Alzheimer Disease Cooperative Study–Activities of Daily Living score (−3.32 [95% CI, −4.02 to −2.62] for rotigotine and −7.24 [95% CI, −7.84 to −6.64] for placebo) and Frontal Assessment Battery score (0.48 [95% CI, 0.31 to 0.65] for rotigotine and −0.66 [95% CI, −0.80 to −0.52] for placebo). There was no longitudinal change in Neuropsychiatric Inventory scores (1.64 [95% CI, 1.06-2.22] for rotigotine and 1.26 [95% CI, 0.77-1.75] for placebo group). Neurophysiological analysis of electroencephalography results indicated that prefrontal cortical activity increased in rotigotine but not in the placebo group. Adverse events were more common in the rotigotine group, with 11 patients dropping out compared with 5 in the placebo group.

**Conclusions and Relevance:**

In this randomized clinical trial, rotigotine treatment did not significantly affect global cognition in patients with mild to moderate AD; however, improvement was observed in cognitive functions highly associated with the frontal lobe and in activities of daily living. These findings suggest that treatment with the dopaminergic agonist rotigotine may reduce symptoms associated with frontal lobe cognitive dysfunction and thus may delay the impairment of activities of daily living.

**Trial Registration:**

ClinicalTrials.gov Identifier: NCT03250741

## Key Points

**Question:**

Can 24 weeks of treatment with rotigotine modify cognitive functions in patients with mild to moderate Alzheimer disease?

**Findings:**

In this randomized clinical trial including 94 participants, 24 weeks of rotigotine treatment did not significantly modify global cognition; however, the treatment did improve frontal lobe functions and was efficacious in reducing functional impairment compared with placebo.

**Meaning:**

These findings suggest that rotigotine may be useful for improving frontal cognitive functions and activities of daily living in patients with mild to moderate Alzheimer disease.

## Teaser

This randomized clinical trial evaluates whether treatment with the dopaminergic agonist rotigotine has an effect on cognitive function in patients with mild to moderate Alzheimer disease in Italy.

## Introduction

In the last decades, much evidence has strengthened the idea that the impairment of dopaminergic transmission may contribute to cognitive dysfunction in Alzheimer disease.^1,2,3^ Dopamine is a key neuromodulator affecting several distinct steps of synaptic transmission, playing an important role in the control of high cognitive functions, such as memory, learning, and decision-making. Postmortem studies have revealed marked loss of dopamine receptors in the temporal and frontal lobes of brains with Alzheimer disease, suggesting an association between decreased levels of D2-like receptor and Alzheimer disease pathophysiology.^4,5^ These neuropathological findings were confirmed by in vivo investigations with positron emission tomography.^6^ Some early attempts have been carried out using dopaminergic drugs, such as L-dopa^7^ or selegiline,^8^ in samples of patients with Alzheimer disease at different stages of the disease, with some controversial results. More recently, experimental studies in animal models of Alzheimer disease showed that dopaminergic agonists may reduce amyloid deposition and improve memory^9,10^ and that the degeneration of dopaminergic neurons in the ventral tegmental area contributes to memory deficits.^11^ It has also been shown that in the early stages of Alzheimer disease, dopaminergic agonists improve cholinergic transmission^12^ and cortical plasticity^13^ likely by acting on the dopaminergic projections over the frontal cortex.^1^ Taken together, this evidence provides novel implications for therapies based on dopaminergic stimulation in patients with mild to moderate Alzheimer disease. Hence, we hypothesized that therapy with dopaminergic agonists could have a relevant clinical effect on cognitive impairment in patients with Alzheimer disease. We performed a trial to evaluate the efficacy and safety of the dopaminergic agonist rotigotine as adjunctive therapy to standard treatment with acetylcholinesterase inhibitors in patients with mild to moderate Alzheimer disease.

## Methods

### Study Design

Patients were eligible for this phase-2 randomized clinical trial if they had an established diagnosis of probable Alzheimer disease according to National Institute of Neurological and Communicative Disorders and Stroke and the Alzheimer Disease and Related Disorders Association criteria; were aged 50 to 85 years; had a Clinical Dementia Rating^14^ score of 0.5 to 1 (scores range from 0 to 3, with higher scores indicating worse dementia) and Mini Mental State Examination (MMSE) score of 18 to 26 at screening (scores range from 0 to 30), indicating mild to moderate Alzheimer disease; had 1 caregiver; had been treated with acetylcholinesterase inhibitor for at least 6 months; and had undergone a lumbar puncture for cerebrospinal fluid biomarkers analysis for diagnostic purposes.^15^ Patients underwent medical and neurologic evaluations, including magnetic resonance imaging or computed tomography. Patients were excluded if they had extrapyramidal signs, history of stroke, another neurodegenerative disorder, psychotic disorders, or if they had been treated within 6 months before enrollment with antipsychotic, antiparkinsonian, anticholinergic, or antiepileptic drugs. The trial was approved by the review board and ethics committee at Santa Lucia Foundation and was conducted in accordance with the principles of the Declaration of Helsinki and the International Conference on Harmonisation Good Clinical Practice guidelines. All patients or their legal representatives provided written informed consent. Patients could withdraw at any point without prejudice. This report followed the Consolidated Standards of Reporting Trials (CONSORT) reporting guideline for randomized studies.

### Randomization and Masking

This was a monocentric, randomized, double-blind trial of rotigotine vs placebo in patients with mild to moderate Alzheimer disease as an add-on to treatment with acetylcholinesterase inhibitors. The trial protocol is available in Supplement 1. The trial comprised a 24-week treatment period with 1 week of dose escalation of transdermal patches of rotigotine at 2 mg per day and 23 weeks of dose maintenance of transdermal patches of rotigotine at 4 mg per day. The dose of rotigotine used in the trial was recommended by an independent data and safety monitoring committee, whose members reviewed data from a safety evaluation and identified the maximum safe dose not associated with unacceptable adverse effects.^16^ A low 4-mg dosage was chosen because such a drug has been previously found to be effective in modulating cholinergic activity and cortical plasticity in patients with Alzheimer disease with no relevant adverse effects.^16^

### Trial Procedures

After recruitment and baseline assessments, patients were randomly assigned in a 1:1 ratio to receive rotigotine or matching placebo in addition to their stable drug regimen with acetylcholinesterase inhibitor therapy. All treatments were administered for 24 weeks with no interruptions. Rotigotine was administered through a 4-mg transdermal patch (Neupro, UCB Pharma) for 23 weeks after administration of a 2-mg patch for 1 week. Transdermal patches of rotigotine had a surface release area of 10 or 20 cm^2^ and contained 4.5 or 9 mg of rotigotine to release, respectively, 2 mg or 4 mg during a 24-hour period when applied to intact skin. The placebo transdermal patch was identical to the rotigotine patch except for the absence of rotigotine. The efficacy assessments were rated at baseline for enrolled patients and caregivers and repeated at week 24 (or upon early termination) by assessors or raters (S.P. and S.B.) who were blinded to the assignment group.

### Outcome Measures

The primary end point was the change at 24 weeks from baseline on the Alzheimer Disease Assessment Scale–Cognitive Subscale (ADAS-Cog-11).^17^ The ADAS-Cog-11 measures severity of impairment in 11 tasks covering various cognitive domains (memory, language, orientation, praxis, and executive functioning). The scale has a score range of 0 to 70 points, with higher scores indicating worse performance. The scale is analyzed as a continuous measure. The intention-to-treat analysis set included all patients who had postbaseline efficacy data. The secondary key end point measures were the change at 24 weeks from baseline on the Activities of Daily Living (ADCS-ADL; scores range from 0 to 78, with higher lower scores indicating worse function),^18^ the Frontal Assessment Battery (FAB; scores range from 0 to 18, with higher scores indicating better frontal cognitive function),^19^ and the Neuropsychiatric Inventory (NPI; scores range from 0 to 144, with higher scores indicating worse behavioral disturbances).^20^ We also used transcranial magnetic stimulation in combination with electroencephalography (TMS-EEG) to monitor the effects of treatment on frontal lobe cortical activity.^21^ We adopted the TMS-EEG approach because it allows for assessment of the neurophysiological state of a specific cortical area. The TMS-EEG approach represents an elective method for the assessment of neural processing through objective measurements of cortical activity in terms of both cortical excitability and oscillatory dynamics.^22^ Hence, we measured as biomarkers neurophysiological changes induced by dopamine-agonist over the left dorsolateral prefrontal cortex (DLPFC) and the left posterior parietal cortex (PPC) by evaluating the cortical excitability and oscillatory activity evoked by single-pulse TMS combined with EEG recordings.^21,22^ For each patient, 80 single TMS pulses were applied over each stimulation site (left DLPFC and left PPC) during an EEG recording with open eyes, with an intensity of 90% of the resting motor threshold. We used TMS-compatible EEG equipment (BrainAmp 32MRpluls, BrainProducts GmbH) to record the EEG activity from 29 scalp sites positioned according to the 10-20 International System. Transcranial magnetic stimulation–compatible Ag/AgCl pellet electrodes were mounted on an elastic cap, and additional electrodes were used as ground and reference. Eye movements were detected by recording an electrooculogram. The EEG and electrooculogram signals were band-pass filtered at 0.1–1000 Hz and digitized at a sampling rate of 5 kHz. Skin and electrode impedance was maintained below 5 kΩ. the TMS-EEG data were analyzed offline (Brain Vision Analyzer, Brain Products GmbH) with different approaches in the spatiotemporal domain for evaluating cortical excitability changes and in the time and frequency domain for evaluating cortical oscillatory changes (for further details, see Supplement 1 and the eMethods in Supplement 2).

Two sets of outcome measures were obtained to assess cortical excitability (global mean field power) and cortical oscillatory activity.^21^ At each clinic visit or upon early termination, adverse events were recorded, vital signs were measured, and physical and neurological examination was performed. An independent data monitoring committee monitored the patients' safety according to the Data Monitoring Committee Charter.

### Statistical Analysis

A total of 94 randomly assigned patients (47 per group) were planned on the basis of a previous study that assessed the effects of rotigotine on cortical plasticity and cognitive functions in a small sample of patients with Alzheimer disease.^13^ In that pilot study, ADAS-Cog-11 data were not collected, however a significant difference was observed in pre-post (12 weeks) treatment with rotigotine in patients in both MMSE and FAB scores. Adopting a power computation based on a 2-tailed paired *t* test, with type I error α = .05 and a plausible correlation between pre-post measured variables of 0.7, the FAB effect size observed in the pilot study equal to 0.42 (obtained as post-pre FAB means over pooled standard deviation) (eAppendix 1 in Supplement 2) requires a minimum sample of 46 participants for reaching a power of 0.8. For MMSE (for which the effect size was 0.48), this sample size allows for reaching a power of 0.9. The minimum total sample size was then augmented up to 92 participants considering the matched placebo group. Randomization was performed and assigned by a statistician (C.F.) working at an independent institution. In order to obtain homogeneous and balanced study groups in terms of age, sex, and *APOE* carriers, an adaptive randomization was adopted^20^ (eAppendix 1 in Supplement 2). Normality assumption of end point variables was assessed by inspection of the distribution plots and by Kolmogorov-Smirnov and Shapiro-Wilk tests (eAppendix 2 in Supplement 2). The longitudinal assessment of the end points across groups was performed through generalized linear mixed models (GLMMs) for repeated measures with random intercept and random slope to account for individual differences at baseline as well as for individual changes during the follow-up.^23^ The GLMMs were applied to ADAS-Cog-11 and to the other efficacy outcome measures, ADCS-ADL, FAB, and NPI, as dependent variables and to “group,” “time,” and “group × time” interaction as independent factors. In detail, GLMMs for Gaussian data with identity link function were applied for ADAS-Cog-11, ADCS-ADL, and FAB, whereas a GLMM for Poisson data with log-link function was used for NPI. The GLMMs on MMSE, ADAS-Cog-11, and FAB were adjusted for age and education (eTable 1 in Supplement 2). To evaluate the treatment effects on TMS-EEG data, we used repeated-measures analysis of variance with between-subjects factor “group” and within-subject factor “time.” All statistical analyses were performed with SPSS Statistics for Windows, version 25 (IBM Corp). Statistical tests were 2-tailed, and *P* < .05 was considered statistically significant.

## Results

**Figure 1.**
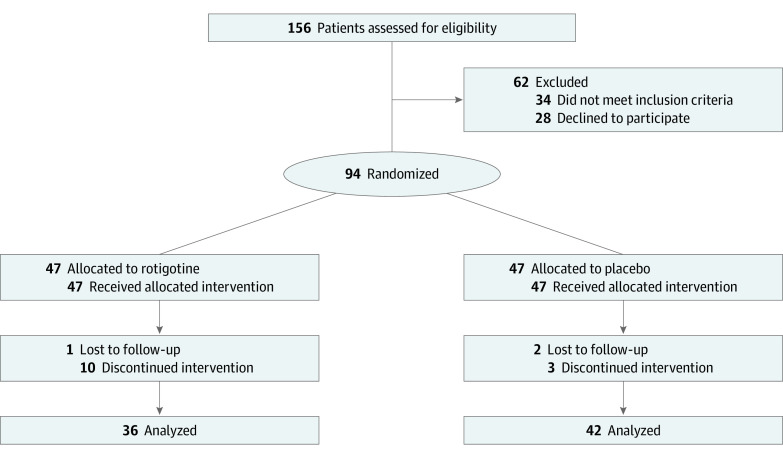
Study Flowchart

A total of 156 patients were screened, and 94 underwent randomization (Figure 1). The mean (SD) age of the total sample of patients was 73.9 (5.6) years (range, 55-83 years), and 58 (62%) were female. Patients had a mean (SD) MMSE raw score at baseline of 23.2 (2.4) points. A total of 58 patients (62%) screened positive as carriers for at least 1 *APOE* ε4 allele. Patients’ baseline demographic and clinical characteristics did not differ between the rotigotine and placebo groups in terms of age; educational level; time since diagnosis of Alzheimer disease; time since current cholinesterase inhibitor treatment initiated; being an *APOE* ε4 carrier; and MMSE, ADAS-Cog-11, FAB, ADCS-ADL, and NPI scores (Table 1). Patients with Alzheimer disease who were enrolled in the present study did not show any significant sign of mild parkinsonism, as confirmed by the Unified Parkinson Disease Rating Scale (UPDRS), Section III (mean [SD] score, 2.6 [1.8] in the rotigotine group and 2.8 [1.6] in the placebo group; scores range from 0 to 56, with worse scores indicating worse motor function) (Table 1). A total of 16 patients withdrew from the trial before completion (11 in the rotigotine group and 5 in the placebo group). A total of 78 patients (83%) completed the treatment period (Figure 1). On the basis of a previous pilot study,^13^ 78 patients were considered enough to reach a power of 0.8 considering an effect size equal to 0.48 for both MMSE and FAB measures.

**Table 1.** Baseline Patients Demographic and Clinical Characteristics at Baseline^a^

Characteristic	Rotigotine group (n = 47)	Placebo group (n = 47)
Age, y	73.4 (5.8)	74.3 (5.5)
Women, No. (%)	31 (66)	27 (57)
Educational attainment, y	8.5 (4.2)	9.4 (4.3)
Time since diagnosis of Alzheimer disease, median (IQR), y	1.3 (0.3-1.9)	1.1 (0.4-1.8)
Time since current cholinesterase inhibitor treatment initiated, median (IQR), y	0.9 (0.6-1.2)	0.8 (0.3-1.1)
*APOE* e4 carrier, No. (%)	28 (60)	30 (64)
MMSE raw score^b^	22.9 (2.3)	23.6 (2.4)
ADAS-Cog-11 raw score^c^	19.8 (6.4)	18.7 (6.5)
FAB raw score^d^	11.4 (3.0)	12.1 (3.0)
ADCS-ADL score^e^	61.0 (12.6)	62.8 (10.4)
NPI score^f^	12.4 (9.9)	12.8 (11.6)
UPDRS III score^g^	2.6 (1.8)	2.8 (1.6)

Abbreviations: ADAS-Cog-11, Alzheimer Disease Assessment Scale–Cognitive Subscale; ADCS-ADL, Alzheimer Disease Cooperative Study–Activities of Daily Living; *APOE*, apolipoprotein E; FAB, Frontal Assessment Battery; IQR, interquartile range; MMSE, Mini Mental State Examination; NPI, Neuropsychiatric Inventory; UPDRS III, Unified Parkinson Disease Rating Scale, Section III.

^a^ Data are presented as mean (SD) unless otherwise indicated.

^b^ Scores range from 0 to 30, with higher scores indicating better cognitive function.

^c^ Scores range from 0 to 70, with higher scores indicating worse cognition.

^d^ Scores range from 0 to 18, with higher scores indicating better frontal cognitive functions.

^e^ Scores range from 0 to 78, with lower scores indicating worse function.

^f^ Scores range from 0 to 144, with higher scores indicating worse behavioral symptoms.

^g^ Scores range from 0 to 56, with higher scores indicating worse motor function.

**Figure 2.**
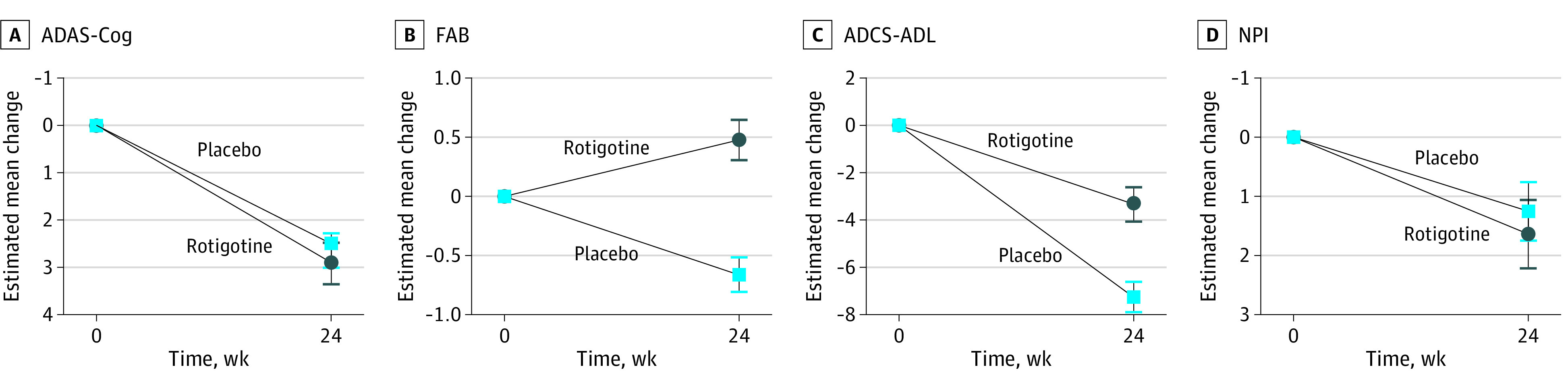
Clinical Data Results A, The generalized linear mixed model estimated mean change from baseline is shown for the Alzheimer Disease Assessment Scale–Cognitive Subscale (ADAS-Cog-11); scores range from 0 to 70, with higher scores indicating worse cognition. B, The estimated mean change from baseline is shown for the Frontal Assessment Battery (FAB); scores range from 0 to 18, with higher scores indicating better frontal cognitive functions. C, The estimated mean change from baseline is shown for the Alzheimer Disease Cooperative Study Activities of Daily Living scale (ADCS-ADL); scores range from 0 to 78, with lower scores indicating worse function. D, The estimated mean change from baseline is shown for the Neuropsychiatric Inventory (NPI); scores range from 0 to 144, with higher scores indicating worse behavioral symptoms. Baseline is plotted at week 0, which is the mean assessment time of the baseline measurement as offset from the first dose of the trial agent. Error bars indicate standard errors.

The mean (SD) baseline ADAS-Cog-11 total score was 19.8 (6.4) for the rotigotine group and 18.7 (6.5) for the placebo group (eFigure 1 in Supplement 2). There were no significant differences at baseline vs week 24 in cognitive performance as measured by the ADAS-Cog-11 total score in the rotigotine group compared with placebo (Table 2). The GLMM for repeated measures on ADAS-Cog-11 scores (adjusted for age and education) did not show any significant result in terms of group effect, time, and time × group interaction, although estimated values showed a general worsening of cognitive performance of patients over time. The GLMM estimated mean change in ADAS-Cog-11 score was 2.92 for the rotigotine group (95% CI, 2.51-3.33) and 2.66 for the placebo group (95% CI, 2.31-3.01) (Figure 2A).

**Table 2.** Change in Primary and Secondary Outcomes From Baseline to Week 24, GLMM Estimated Effects

Outcome	Estimated change from baseline, mean (95% CI)		Group effect		Time effect		Group × time effect	
	Rotigotine	Placebo	*F* value	*P* value	*F* value	*P* value	*F* value	*P* value
Primary outcome								
ADAS-Cog-11 score^a^	2.92 (2.51 to 3.33)	2.66 (2.31 to 3.01)	*F*_1163_ = 0.37	.55	*F*_1163_ = 0.14	.71	*F*_1163_ = 0.05	.82
Secondary outcomes								
ADCS-ADL score	−3.32 (−4.02 to −2.62)	−7.24 (−7.84 to −6.64)	*F*_1164_ = 0.02	.88	*F*_1164_ = 0.17	.68	*F*_1164_ = 4.24	.04
FAB score^a^	0.48 (0.31 to 0.65)	−0.66 (−0.80 to −0.52)	*F*_1164_ = 0.04	.84	*F*_1164_ = 0.001	.98	*F*_1164_ = 5.99	.02
NPI total score^a^	1.64 (1.06 to 2.22)	1.26 (0.77 to 1.75)	*F*_1164_ = 0.01	.93	*F*_1164_ = 0.02	.89	*F*_1164_ = 0.05	.82

Abbreviations: ADAS-Cog-11, The Alzheimer Disease Assessment Scale–Cognitive Subscale; ADCS-ADL, Alzheimer Disease Cooperative Study–Activities of Daily Living; FAB, Frontal Assessment Battery; GLMM, generalized linear mixed model; NPI, Neuropsychiatric Inventory.

^a^ Adjusted for age and education.

The analysis of secondary outcomes showed significant differences between the rotigotine group and the placebo group for the FAB and ADCS-ADL scores but not for the NPI scores (Table 2). The GLMM estimated mean change in FAB score was 0.48 for the rotigotine group (95% CI, 0.31-0.65) and −0.66 for the placebo group (95% CI −0.80 to −0.52), suggesting that frontal lobe functions improved in the rotigotine group compared with the placebo group (*P* = .02 for interaction) (Figure 2B). The baseline mean (SD) of ADCS-ADL total score was 61.0 (12.6) for the rotigotine group and 62.8 (10.1) for the placebo group. Estimated mean change in ADCS-ADL scores was −3.32 for the rotigotine group (95% CI, −4.02 to −2.62) and −7.24 for the placebo group (95% CI, −7.84 to −6.64), showing an advantage of the rotigotine treatment compared with placebo (*P* = .04 for interaction) (Figure 2C). The baseline mean (SD) for NPI total score was 12.4 (9.9) for the rotigotine group and 12.8 (11.6) for the placebo group. Estimated mean change in NPI score was 1.64 for the rotigotine group (95% CI, 1.06-2.22) and 1.26 for the placebo group (95% CI, 0.77-1.75), suggesting no significant effects between the groups during follow-up (Figure 2D and eTable 2 in Supplement 2).

**Figure 3.**
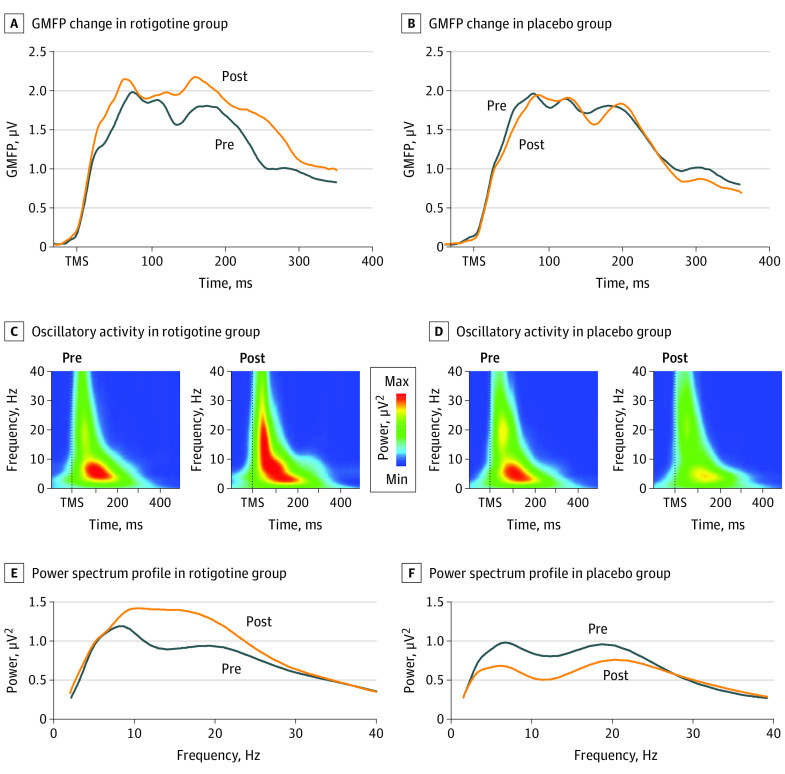
TMS-EEG Results Changes in global mean field power (GMFP) (A, B) and oscillatory activity (C-F) evoked from the left dorsolateral prefrontal cortex in the rotigotine and placebo groups before and after completion of the trial. The upper panels depict the electroencephalographic activity evoked by transcranial magnetic simulation (TMS) before and after the 24-week period of treatment with rotigotine (A) or placebo (B). The middle panels show changes in oscillatory activity in the group of patients treated with rotigotine (C) and placebo (D), with dark blue indicating lower oscillatory activity; intense red, stronger oscillatory activity; and green, an intermediate value. Panels E and F show the power spectrum profile of evoked oscillatory activity depicted in panels C and D.

After 24 weeks of treatment, we observed a significant increase of DLPFC activity as measured by global mean field power in the rotigotine group (20 patients) as compared with the placebo group (20 patients) (treatment × time interaction, *F*_1,38_ = 11.235; *P* = .002). We also found a significant increase of DLPFC oscillatory activity in the rotigotine group as compared with the placebo group (treatment × time interaction, *F*_1,38_ = 6.837; *P* = .01) (Figure 3). This effect was site specific, because no change in cortical activity was observed when TMS pulses were applied over the PPC (eFigure 2 in Supplement 2).

In the current trial, adverse events were more common with rotigotine than with placebo. In total, 16 patients dropped out, 11 of whom were assigned to rotigotine treatment and 5 to placebo. In the rotigotine group, 2 patients reported allergy to the patch, 1 had visual hallucinations, 1 had pneumonia, 3 had nausea and dizziness, 1 had sleep disorders, 1 had anxiety, 1 was implanted with a cardiac pacemaker, and 1 declined to continue. In the placebo group, 1 patient had pneumonia, 1 had cervical pain, 1 had a diagnosis of kidney tumor, 2 refused to continue.

## Discussion

This randomized clinical trial presents the results of a dopamine-agonist treatment with rotigotine in patients with mild to moderate Alzheimer disease. In this trial, a daily dose of rotigotine showed no benefit with respect to the primary clinical outcome as measured by change in the ADAS-Cog-11 score from baseline to week 24 as compared with placebo. Nevertheless, our results showed that rotigotine at a relatively low dosage was safe and well tolerated in patients with mild to moderate Alzheimer disease. Adverse events were more common with rotigotine than with placebo but were similar to those seen in randomized controlled trials testing rotigotine in patients with mild Parkinson disease of comparable duration.^24,25^ Moreover, rotigotine did not induce any relevant behavioral side effects as revealed by NPI scores analysis. Notably, patients enrolled in the current study were in the early phase of Alzheimer disease and did not show any extrapyramidal signs, such as tremor or rigidity.^26^ In agreement with previous studies showing that extrapyramidal symptoms are more likely to appear in the later stages of Alzheimer disease, patients enrolled in the present study did not show a significant rate of mild parkinsonism at the earlier stages of Alzheimer disease, as confirmed by the UPDRS assessment (Table 1).

The primary outcome analysis showed that rotigotine administration had no effects on memory and other cognitive tasks, as measured by ADAS-Cog-11. However, secondary outcome analysis showed a clear and remarkable effect on cognitive functions highly related to the frontal lobe. We chose to evaluate the effects of rotigotine on frontal lobe functions because dopamine largely modulates frontal cortex activity,^27^ and a previous study showed that treatment with rotigotine induces an improvement of cortical plasticity in the frontal cortex in patients with mild Alzheimer disease.^13^

In the present trial, we found that rotigotine improved cognitive functions highly related to the frontal lobe in patients with Alzheimer disease during 24 weeks, while these cognitive functions declined in patients treated with placebo. Moreover, rotigotine was efficacious in reducing the decline of functional impairment. Our study showed an effect on the activities of daily living in the rotigotine group compared with the placebo group, suggesting that use of rotigotine could have a potential role in treating functional impairment starting in the early stages of the disease.

In addition to memory impairment, a decline in cognitive functions related to the frontal lobe activity and in everyday living activities represent the key features of Alzheimer disease progression.^28^ Executive functions play a crucial role in coping with the changing demands of everyday life and are associated with frontal lobe activity.^29^ The preservation of everyday living activities is closely related to executive functions, and their impairment is associated with early loss of independence, shifting many daily responsibilities to caregivers and increasing their burden.^30^ In this regard, the impairment of everyday living activities in patients with Alzheimer disease has been associated with global pathologic changes and frontal hypometabolism.^31,32^ Therefore, treating frontal cognitive impairment should be one of the main targets for future pharmacological interventions.

Apart from the positive effects on cognitive functions highly related to the frontal lobe, we also found that rotigotine induced a remarkable increase of prefrontal cortex activity, as indexed by TMS-EEG recordings. Treatment with rotigotine also enhanced the evoked EEG response to TMS, resulting in increased oscillatory activity in the range of alfa and beta frequencies.

Prolonged exposure to amyloid beta protein progressively impairs the physiological release of dopamine in the prefrontal cortex and hippocampus, contributing to the impairment of attention, memory, and executive functions.^33,34^ Magnetic resonance imaging recently showed that volume and connectivity of the ventral tegmental area are associated with cognitive impairment in patients with mild Alzheimer disease.^35^ Notably, the ventral tegmental area is the major source of dopaminergic projections directed toward the prefrontal cortex through mesocortical fibers.^36^ In agreement with this background, our combined clinical and TMS-EEG findings suggest that increasing dopaminergic neurotransmission with rotigotine likely enhances frontal lobe activity by acting on mesocortical dopaminergic projections.

### Limitations

This study has some limitations. Despite the improvement of cognitive functions highly related to the frontal lobe, we did not observe any effect on memory, as also revealed by the analysis of ADAS-Cog-11 subitems. It is possible that the association between dopamine agonists and cholinesterase inhibitors could have masked measurable effects on memory tasks.^37^ On the other hand, the medial temporal lobe is a site of complex pathological mechanisms linking neurodegeneration with neuroinflammation^38^ that likely begin long before cognitive decline appears, making the contribution of dopaminergic neurotransmission negligible in patients with moderate Alzheimer disease. Moreover, owing to the relative low number of patients enrolled, our study did not take into account the potential influence of *APOE* genotype and cognitive reserve. Further studies are needed to clarify these issues.

## Conclusions

Currently, no cure or disease-modifying treatment is available for Alzheimer disease, and recent attempts with novel disease-modifying drugs have been ineffective.^39,40,41^ The most frequently prescribed treatments for Alzheimer disease are acetylcholinesterase inhibitors and memantine.^42^ In addition, the current treatments are not effective for everyone; it is estimated that approximately 40% to 70% of patients benefit from current treatments. Given the significant limitations of the current treatment options, more effective symptomatic therapies, particularly in the earlier stages of Alzheimer disease, are needed.

Within this framework, the present randomized clinical trial indicates that the use of dopaminergic agonists, such as rotigotine, is safe in patients with mild to moderate Alzheimer disease. Treatment with rotigotine may have a potential effect in reducing symptoms associated with frontal lobe cognitive dysfunction and in delaying the impairment of activities of daily living.

## Notes

Supplement 1. **Trial Protocol**

Supplement 2. **eAppendix 1.** Supplementary Statistics of Clinical Data **eAppendix 2.** Supplementary Statistics of TMS-EEG Data **eFigure 1.** Box Plot of Clinical Variables **eFigure 2.** Global Mean Field Power and Oscillatory Activity Evoked From the Left Posterior Parietal Cortex in the Rotigotine and Placebo Groups Before and After Completion of the Trial **eTable 1.** Association Between Age, Education and Secondary End Points Evaluated by Linear Regression Model for MMSE, ADAS, FAB, ADCS-ADL and Poisson Regression for NPI **eTable 2.** Generalized Linear Mixed Model With Poisson Distribution for NPI Subscales **eMethods.** Supplementary Methods for TMS-EEG Recordings and Processing **eReferences.**

Supplement 3. **Data Sharing Statement**

## References

[1] Martorana A, Koch G Is dopamine involved in Alzheimer’s disease? Front Aging Neurosci. 2014;6:252. doi:10.3389/fnagi.2014.00252 25309431 PMC4174765

[2] Itoh A, Nitta A, Nadai M, Dysfunction of cholinergic and dopaminergic neuronal systems in beta-amyloid protein–infused rats. J Neurochem. 1996;66(3):1113-1117. doi:10.1046/j.1471-4159.1996.66031113.x 8769873

[3] Joyce JN, Smutzer G, Whitty CJ, Myers A, Bannon MJ Differential modification of dopamine transporter and tyrosine hydroxylase mRNAs in midbrain of subjects with Parkinson’s, Alzheimer’s with parkinsonism, and Alzheimer’s disease. Mov Disord. 1997;12(6):885-897. doi:10.1002/mds.870120609 9399211

[4] Pan X, Kaminga AC, Wen SW, Wu X, Acheampong K, Liu A Dopamine and dopamine receptors in Alzheimer’s disease: a systematic review and network meta-analysis. Front Aging Neurosci. 2019;11:175. doi:10.3389/fnagi.2019.00175 31354471 PMC6637734

[5] Kumar U, Patel SC Immunohistochemical localization of dopamine receptor subtypes (D1R-D5R) in Alzheimer’s disease brain. Brain Res. 2007;1131(1):187-196. doi:10.1016/j.brainres.2006.10.049 17182012

[6] Kemppainen N, Laine M, Laakso MP, Hippocampal dopamine D2 receptors correlate with memory functions in Alzheimer’s disease. Eur J Neurosci. 2003;18(1):149-154. doi:10.1046/j.1460-9568.2003.02716.x 12859348

[7] Lewis C, Ballinger BR, Presly AS Trial of levodopa in senile dementia. BMJ. 1978;1(6112):550. doi:10.1136/bmj.1.6112.550 343866 PMC1603209

[8] Birks J, Flicker L Selegiline for Alzheimer’s disease. Cochrane Database Syst Rev. 2003;(1):CD000442. 12535396 10.1002/14651858.CD000442

[9] Himeno E, Ohyagi Y, Ma L, Apomorphine treatment in Alzheimer mice promoting amyloid-β degradation. Ann Neurol. 2011;69(2):248-256. doi:10.1002/ana.22319 21387370

[10] Moreno-Castilla P, Rodriguez-Duran LF, Guzman-Ramos K, Barcenas-Femat A, Escobar ML, Bermudez-Rattoni F Dopaminergic neurotransmission dysfunction induced by amyloid-β transforms cortical long-term potentiation into long-term depression and produces memory impairment. Neurobiol Aging. 2016;41:187-199. doi:10.1016/j.neurobiolaging.2016.02.021 27103531

[11] Nobili A, Latagliata EC, Viscomi MT, Dopamine neuronal loss contributes to memory and reward dysfunction in a model of Alzheimer’s disease. Nat Commun. 2017;8:14727. doi:10.1038/ncomms14727 28367951 PMC5382255

[12] Martorana A, Mori F, Esposito Z, Dopamine modulates cholinergic cortical excitability in Alzheimer’s disease patients. Neuropsychopharmacology. 2009;34(10):2323-2328. doi:10.1038/npp.2009.60 19516251

[13] Koch G, Di Lorenzo F, Bonnì S, Dopaminergic modulation of cortical plasticity in Alzheimer’s disease patients. Neuropsychopharmacology. 2014;39(11):2654-2661. doi:10.1038/npp.2014.119 24859851 PMC4207345

[14] Morris JC The Clinical Dementia Rating (CDR): current version and scoring rules. Neurology. 1993;43(11):2412-2414. doi:10.1212/WNL.43.11.2412-a 8232972

[15] Dubois B, Feldman HH, Jacova C, Advancing research diagnostic criteria for Alzheimer’s disease: the IWG-2 criteria. Lancet Neurol. 2014;13(6):614-629. doi:10.1016/S1474-4422(14)70090-0 24849862

[16] Martorana A, Di Lorenzo F, Esposito Z, Dopamine D_2_-agonist rotigotine effects on cortical excitability and central cholinergic transmission in Alzheimer’s disease patients. Neuropharmacology. 2013;64:108-113. doi:10.1016/j.neuropharm.2012.07.015 22863599

[17] Mohs RC, Knopman D, Petersen RC, Development of cognitive instruments for use in clinical trials of antidementia drugs: additions to the Alzheimer’s Disease Assessment Scale that broaden its scope. The Alzheimer’s Disease Cooperative Study. Alzheimer Dis Assoc Disord. 1997;11(suppl 2):S13-S21. doi:10.1097/00002093-199700112-00003 9236948

[18] Galasko D, Bennett D, Sano M, An inventory to assess activities of daily living for clinical trials in Alzheimer’s disease. The Alzheimer’s Disease Cooperative Study. Alzheimer Dis Assoc Disord. 1997;11(suppl 2):S33-S39. doi:10.1097/00002093-199700112-00005 9236950

[19] Dubois B, Slachevsky A, Litvan I, Pillon B The FAB: a Frontal Assessment Battery at bedside. Neurology. 2000;55(11):1621-1626. doi:10.1212/WNL.55.11.1621 11113214

[20] Cummings JL, Mega M, Gray K, Rosenberg-Thompson S, Carusi DA, Gornbein J The Neuropsychiatric Inventory: comprehensive assessment of psychopathology in dementia. Neurology. 1994;44(12):2308-2314. doi:10.1212/WNL.44.12.2308 7991117

[21] Koch G, Bonnì S, Casula EP, Effect of cerebellar stimulation on gait and balance recovery in patients with hemiparetic stroke: a randomized clinical trial. JAMA Neurol. 2019;76(2):170-178. doi:10.1001/jamaneurol.2018.3639 30476999 PMC6439971

[22] Kumar S, Zomorrodi R, Ghazala Z, Extent of dorsolateral prefrontal cortex plasticity and its association with working memory in patients with Alzheimer disease. JAMA Psychiatry. 2017;74(12):1266-1274. doi:10.1001/jamapsychiatry.2017.3292 29071355 PMC6583382

[23] Lin J, Lin LA, Sankoh S A general overview of adaptive randomization design for clinical trials. J Biom Biostat. 2016;7(2):294. doi:10.4172/2155-6180.1000294

[24] Poewe WH, Rascol O, Quinn N, ; SP 515 Investigators Efficacy of pramipexole and transdermal rotigotine in advanced Parkinson’s disease: a double-blind, double-dummy, randomised controlled trial. Lancet Neurol. 2007;6(6):513-520. doi:10.1016/S1474-4422(07)70108-4 17509486

[25] LeWitt PA, Lyons KE, Pahwa R; SP 650 Study Group Advanced Parkinson disease treated with rotigotine transdermal system: PREFER Study. Neurology. 2007;68(16):1262-1267. doi:10.1212/01.wnl.0000259516.61938.bb 17438216

[26] Park JH, Myung W, Choi J, Extrapyramidal signs and cognitive subdomains in Alzheimer disease. Am J Geriatr Psychiatry. 2016;24(7):566-574. doi:10.1016/j.jagp.2016.02.051 27067071

[27] Ott T, Nieder A Dopamine and cognitive control in prefrontal cortex. Trends Cogn Sci. 2019;23(3):213-234. doi:10.1016/j.tics.2018.12.006 30711326

[28] Liu-Seifert H, Siemers E, Price K, ; Alzheimer’s Disease Neuroimaging Initiative Cognitive impairment precedes and predicts functional impairment in mild Alzheimer’s disease. J Alzheimers Dis. 2015;47(1):205-214. doi:10.3233/JAD-142508 26402769 PMC4923754

[29] Schroeter ML, Vogt B, Frisch S, Executive deficits are related to the inferior frontal junction in early dementia. Brain. 2012;135(Pt 1):201-215. doi:10.1093/brain/awr311 22184615 PMC3267982

[30] Marshall GA, Rentz DM, Frey MT, Locascio JJ, Johnson KA, Sperling RA; Alzheimer’s Disease Neuroimaging Initiative Executive function and instrumental activities of daily living in mild cognitive impairment and Alzheimer’s disease. Alzheimers Dement. 2011;7(3):300-308. doi:10.1016/j.jalz.2010.04.005 21575871 PMC3096844

[31] Salmon E, Lespagnard S, Marique P, Cerebral metabolic correlates of four dementia scales in Alzheimer’s disease. J Neurol. 2005;252(3):283-290. doi:10.1007/s00415-005-0551-3 16189724

[32] Rowe CC, Ng S, Ackermann U, Imaging beta-amyloid burden in aging and dementia. Neurology. 2007;68(20):1718-1725. doi:10.1212/01.wnl.0000261919.22630.ea 17502554

[33] Trillo L, Das D, Hsieh W, Ascending monoaminergic systems alterations in Alzheimer’s disease. translating basic science into clinical care. Neurosci Biobehav Rev. 2013;37(8):1363-1379. doi:10.1016/j.neubiorev.2013.05.008 23707776

[34] Duszkiewicz AJ, McNamara CG, Takeuchi T, Genzel L Novelty and dopaminergic modulation of memory persistence: a tale of two systems. Trends Neurosci. 2019;42(2):102-114. doi:10.1016/j.tins.2018.10.002 30455050 PMC6352318

[35] De Marco M, Venneri A Volume and connectivity of the ventral tegmental area are linked to neurocognitive signatures of Alzheimer’s disease in humans. J Alzheimers Dis. 2018;63(1):167-180. doi:10.3233/JAD-171018 29578486

[36] Morales M, Margolis EB Ventral tegmental area: cellular heterogeneity, connectivity and behaviour. Nat Rev Neurosci. 2017;18(2):73-85. doi:10.1038/nrn.2016.165 28053327

[37] Takeuchi T, Duszkiewicz AJ, Sonneborn A, Locus coeruleus and dopaminergic consolidation of everyday memory. Nature. 2016;537(7620):357-362. doi:10.1038/nature19325 27602521 PMC5161591

[38] Alcolea D, Martínez-Lage P, Sánchez-Juan P, Amyloid precursor protein metabolism and inflammation markers in preclinical Alzheimer disease. Neurology. 2015;85(7):626-633. doi:10.1212/WNL.0000000000001859 26180139

[39] Doody RS, Thomas RG, Farlow M, ; Alzheimer’s Disease Cooperative Study Steering Committee; Solanezumab Study Group Phase 3 trials of solanezumab for mild-to-moderate Alzheimer’s disease. N Engl J Med. 2014;370(4):311-321. doi:10.1056/NEJMoa1312889 24450890

[40] Salloway S, Sperling R, Fox NC, ; Bapineuzumab 301 and 302 Clinical Trial Investigators Two phase 3 trials of bapineuzumab in mild-to-moderate Alzheimer’s disease. N Engl J Med. 2014;370(4):322-333. doi:10.1056/NEJMoa1304839 24450891 PMC4159618

[41] Egan MF, Kost J, Tariot PN, Randomized trial of verubecestat for mild-to-moderate Alzheimer’s disease. N Engl J Med. 2018;378(18):1691-1703. doi:10.1056/NEJMoa1706441 29719179 PMC6776074

[42] Howard R, McShane R, Lindesay J, Donepezil and memantine for moderate-to-severe Alzheimer’s disease. N Engl J Med. 2012;366(10):893-903. doi:10.1056/NEJMoa1106668 22397651

