# Error in Affiliations and Corresponding Author Email

**DOI:** 10.1001/jamanetworkopen.2020.19190

**Published:** 2020-08-18

In the Original Investigation titled “Effect of Rotigotine vs Placebo on Cognitive Functions Among Patients With Mild to Moderate Alzheimer Disease: A Randomized Clinical Trial,”^1^ published online July 15, 2020, the email address for corresponding author Alessandro Martorana was written incorrectly, and author Francesco Di Lorenzo’s affiliations incorrectly included the Unit of Statistics, IRCCS Istituto Centro San Giovanni di Dio Fatebenefratelli, Brescia, Italy. This article has been corrected.^1^
