# Supplementary material for: Effect of Rotigotine vs Placebo on Cognitive Functions Among Patients With Mild to Moderate Alzheimer Disease: A Randomized Clinical Trial
Source: JAMA Netw Open. 2020 Jul 15;3(7):e2010372. doi: 10.1001/jamanetworkopen.2020.10372 (PMC7364345; doi:10.1001/jamanetworkopen.2020.10372)
Supplement: Supplement 1. — Trial Protocol [file jamanetwopen-3-e2010372-s001.pdf]

Protocol and statistical analysis plan for: Koch G, Motta C, Bonni S, et al. A randomized trial with dopaminergic agonist rotigotine for Alzheimer's disease.

This supplement contains the following items:

1. STUDY PROTOCOL

1.1 ADMINISTRATIVE INFORMATION…………………………..........page 2
1.2 SYNOPSIS…………………………………………………..........page 3
1.3 ABBREVIATIONS AND DEFINITIONS……………………….........page 5
1.4 RATIONALE……………………………………………….........page 7
1.5 OBJECTIVES………………………………………………………page 9
1.6 INVESTIGATIONAL PLAN…………………………………………page 10
1.7 STUDY POPULATION……………………………………….....page 14
1.8 TREATMENT……………………………………………........page 16
1.9 EFFICACY AND SAFETY EVALUATIONS:
OUTCOME MEASURES……………………………………………page 18
1.10 INFORMED CONSENT, ETHICAL REVIEW AND REGULATORY CONSIDERATIONS…………………………………………………page 20

2. STATISTICAL ANALYSIS PLAN

2.1 SAMPLE SIZE AND STATISTICAL METHODS……………………page 23

REFERENCES………………………………………………………..page 26

1. STUDY PROTOCOL

SECTION 1: ADMINISTRATIVE INFORMATION

Title: Effects of Dopaminergic Therapy in Patients With Alzheimer's Disease: a Phase II 24-week, Randomized, Double-blind Placebo Controlled Study.

Trial registration number: ClinicalTrials.gov Identifier: NCT03250741

Sap version: V1

Protocol version: V1

SAP roles and responsibility: Giacomo Koch (P. I.), Clarissa Ferrari (statistician).

SECTION 2: SYNOPSIS

Recent experimental works reveal that the dopaminergic (DA) system may well be involved in the occurrence of cognitive decline, often being predictive of rapidly progressive forms of Alzheimer's disease (AD). In association to cognitive decline symptoms about 35-40% of AD patients present with extrapyramidal signs, supporting the idea that DA-containing neurons undergo degenerative changes. In AD, neurons forming the nigrostriatal pathway show several pathologic changes like neurofibrillary tangles, Aβ plaques, neuropil threads, neuronal loss and also decrease in DA content, all changes suggesting the clear involvement of DA in the pathophysiology of cognitive decline and non-cognitive symptoms of AD. Reduced expression of both subtypes of DA receptors, D1-like and D2-like has been observed in prefrontal cortex and in hippocampus of AD patients. Interestingly, although the dorsal striatum is relatively spared in AD, its ventral homologous, the nucleus accumbens, is highly affected. Furthermore, recent imaging studies showed atrophy of this nucleus in a cohort of late onset AD patients, but not in the early-onset AD patients. Recent works from our group showed surprising positive effects of DA drugs on cortical neurotransmission, synaptic plasticity mechanisms and also on cognitive performances, suggesting a possible therapeutic effects for these drugs in the treatment of AD. The primary objective of this protocol is to verify the efficacy on cognition and overall clinical response of dopaminergic-agonist rotigotine, a cognitive enhancer that improves cholinergic activity and cortical plasticity, in patients with mild AD. Specifically, the main aim is to determine if treatment with a common FDA-approved dopaminergic agonist-rotigotine, as add-on therapy to cholinesterase inhibitors treatment (donepezil or rivastigmine), can improve cognitive abilities and modify physiological cortical activity in patients with mild AD. Rotigotine treatment in add-on therapy was used in our previous experience (Koch et al., 2014), and was well tolerated by patients with no side effects. To test if rotigotine changes brain physiology, we will use neurophysiological tools such as transcranial magnetic stimulation and electroencephalography to measure changes in cortical activity. This is a phase IIa 24-week,

prospective, randomized, double-blind placebo controlled study. The study is designed to evaluate the efficacy, safety, and tolerability of transdermal patch of Rotigotine (RTG) versus placebo (PLC) as add-on therapy with AChEI in patients with mild AD according to the consensus diagnostic criteria and MMSE score of ≥18 and ≤24 at screening. Two groups of patients with mild AD will be involved (50 patients each). One group will be assigned to treatment with RTG 4 mg and the other one to PLC as add on to AChEI therapy. Clinical and neurophysiological measurements will be collected before and after drug administration.

SECTION 3. ABBREVIATIONS AND DEFINITIONS

Aβ: amyloid- β peptides

AChEI: acetylcholinesterase inhibitor

AD: Alzheimer's disease

ADAS-Cog: Alzheimer's Disease Assessment Scale–Cognitive subscale

ADCS-ADL: Alzheimer's Disease Cooperative Study–Activities of Daily Living Inventory

ADDF: Alzheimer's Drug Discovery Foundation

AE: adverse event: Any untoward medical occurrence in a patient or clinical investigation subject administered a pharmaceutical product that does not necessarily have a causal relationship with this treatment. An adverse event can therefore be any unfavorable and unintended sign (including an abnormal laboratory finding), symptom, or disease temporally associated with the use of a medicinal (investigational) product, whether or not related to the medicinal (investigational) product.

ANOVA: analysis of variance

APOE: apolipoprotein E

Blinding: A procedure in which one or more parties to the trial are kept unaware of the treatment assignment(s). Blinding will remain in effect until final database lock. A double-blind study is one in which neither the patient nor any of the investigator who are involved in the treatment or clinical evaluation of the subjects are aware of the treatment received.

CDR: Clinical Dementia Rating Scale

CRF: Case report form, a printed or electronic form for recording study patients' data during a clinical study, as required by the protocol.

CSF: cerebrospinal fluid

DA: dopamine

EEG: electroencephalogram

Efficacy: Efficacy is the ability of a treatment to achieve a beneficial intended result under controlled conditions.

End of study (trial): The date of the last visit or last scheduled procedure shown in the Study. Schedule for the last active patient in the study.

FAB: Frontal assessment battery

GABA: Gamma-Aminobutyric acid

GLMM: Generalized Linear Mixed Model

Informed consent: A process by which a patient voluntarily confirms his or her willingness to participate in a particular trial, after having been informed of all aspects of the trial that are relevant to the patient's decision to participate. Informed consent is documented by means of a written, signed, and dated informed consent form.

Intent-to-Treat (ITT): The principle that asserts that the effect of a treatment policy can be best assessed by evaluating on the basis of the intention to treat a patient (that is, the planned treatment regimen) rather than

the actual treatment given. It has the consequence that patients allocated to a treatment group should be followed up, assessed, and analyzed as members of that group, irrespective of their compliance to the planned course of treatment.

Investigational product: A pharmaceutical form of an active ingredient or placebo being tested or used as a reference in a clinical trial

Investigator: A person responsible for the conduct of the clinical study at a study site. If a study is conducted by a team of individuals at a study site, the investigator is the responsible leader of the team and may be called the principal investigator

IRB/ERB: Institutional Review Board/Ethical Review Board: a board or committee (institutional, regional, or national) composed of medical professional and nonmedical members whose responsibility is to verify that the safety, welfare, and human rights of the patients participating in a clinical study are protected.

MMSE: Mini-Mental State Examination

NINCDS/ADRDA: National Institute of Neurological and Communicative Disorders and Stroke/Alzheimer's Disease and Related Disorders Association

NPI: Neuropsychiatric Inventory

Patient: A study participant who has the disease or condition for which the investigational product is targeted.

PFC: dorsolateral prefrontal cortex

SAP: statistical analysis plan

Study Entry Terms:

1) Screen: The act of determining if an individual meets minimum requirements to become part of a pool of potential candidates for participation in a clinical study. In this study, screening involves medical and neurological tests, including magnetic resonance imaging or computed tomography, and lumbar puncture for CSF biomarkers analysis for diagnostic purposes.

2) Enroll: The act of assigning a patient to a treatment. Patients who are enrolled in the study are those who have been assigned to a treatment.

3) Enter: The act of obtaining informed consent for participation in a clinical study from patients deemed eligible or potentially eligible to participate in the clinical study. Patients entered into a study are those who sign the informed consent form directly or through their legally acceptable representatives.

TMS: Transcranial Magnetic Stimulation.

SECTION 4: STUDY RATIONALE

In recent years several evidences suggested that the impairment of synaptic plasticity represents a pathogenic key in developing AD. Dopamine (DA) is a key neuromodulator affecting several distinct steps of synaptic transmission, including the probability of neurotransmitter release, the post-synaptic sensitivity to neurotransmitter, and the membrane excitability of the pre- and post-synaptic cells. From a cognitive perspective, DA is known to play an important role in the control of high cognitive functions such as memory, learning, attention and decision making. There is experimental evidence demonstrating that in the cerebral cortex, as well as in the basal forebrain, DA modulates the activity not only of pyramidal cells and GABA interneurons, but also of diffuse cholinergic projections from neurons located in the basal forebrain (Goldman-Rakic et al.,1999, and 2000; Paspalas et al., 2005; Berlanga et al., 2005; Zhang et al., 2009). Notably, the dysfunction of dopaminergic transmission has been hypothesized as a new player in the pathophysiology of Alzheimer's disease (Itoh et al., 1996; Joyce et al., 1998; Kemppainen et al., 2003; Kumar and Patel, 2007; Martorana et al., 2010, Martorana and Koch, 2014). Post mortem studies revealed marked loss of DA receptors in the temporal and frontal lobes of Alzheimer's disease brains (Itoh et al., 1996; Kemppainen et al., 2003; Kumar and Patel., 2007; Martorana et al., 2010), regions classically involved in cognitive decline. Interestingly, most of changes regarding DA receptors, particularly D2 subtype, were found to be largely reduced at rostral and mid-levels of the temporal cortex, indicating that regions classically affected by Alzheimer's disease pathology are also sensitive to the loss of D2 receptors (Goldsmith et al., 1996; Joyce et al., 1993, 1998a; Ryoo et al., 1994). These dopamine D2/D3 receptors may play an important role in the reciprocal activity of large groups of neurons in the high-order association cortical regions, and may promote the cognitive and behavioural impairments observed in Alzheimer's disease (Joyce et al., 1998b). D2 receptor binding was significantly reduced in the striatum of Alzheimer's disease patients, even in the absence of overt extra-pyramidal symptoms (Pizzolato et al., 1996). Kemppainen and colleagues (2003) reported that D2 receptor binding potentials are reduced in the hippocampus by 30% in

Alzheimer’s disease patients as compared to controls. This reduction was found to be associated with both cognitive (Kemppainen et al., 2003) and behavioral abnormalities in Alzheimer’s disease patients (Tanaka et al., 2003). Localization studies of dopamine receptors in Alzheimer’s disease brains have shown a preferential reduction of D2-like receptors in the hippocampus and prefrontal cortex (Kemppainen et al., 2003; Kumar and Patel, 2007). Prolonged exposure to Aβ would progressively impair the physiological release of glutamate and of GABA reducing the possibility of DA release in prefrontal cortex and hippocampus, contributing to the impairment of attention, memory, and executive functions. Our group performed a series of neurophysiological studies using transcranial magnetic stimulation (TMS) in order to investigate possible modulatory effects of DA on cortical synaptic transmission and plasticity in AD. We first demonstrated that cholinergic transmission, as measured by short-latency afferent inhibition (SLAI) TMS protocol (Di Lazzaro et al., 2002), can be transiently restored by the acute administration of a single dose of l-dopa (Martorana et al., 2009). Similar findings can be obtained by applying transdermal patches of rotigotine, which is a DA D2/D3-agonist (Martorana et al., 2013). Moreover, we discovered that that treatment with dopamine agonist rotigotine restored the altered mechanism of LTP-like cortical plasticity in Alzheimer’s disease patients (Koch et al., 2012; Koch et al., 2014). Crucially, this study revealed that DA agonists seem to have some positive effects on general cognitive functions (as shown by an increase of the MMSE) and more specifically on frontal executive functions (as revealed by increased scores in the FAB), paving the way to clinical trial based on dopaminergic therapy. This hypothesis finds support on recent experimental studies showing that in animal models AD dopamine agonists may improve memory and even reduce intraneuronal amyloid deposition (Guzmán-Ramos et al., 2012; Himeno et al., 2011). The current project has the ambition to provide first time evidence that dopaminergic stimulation may have a clinical impact in patients with mild AD. Neurophysiological investigations will allow us to identify quantifiable biomarkers underlying the effects induced by dopamine agonist on the neurodegenerative brain. The application of recent neurophysiological tools, such as the combined use of transcranial magnetic stimulation

(TMS) during electroencephalography (EEG) will allow us to understand how dopamine agonists are able to modulate the cortical activity of the prefrontal cortex in AD patients (Kähkönen et al., 2005; Julkunen et al., 2008), likely trough DA terminals originating from the ventral tegmental nucleus, defining the neurophysiological biomarkers of clinical improvement.

SECTION 5: OBJECTIVES

The study is designed to evaluate the efficacy, safety, and tolerability of transdermal patch of Rotigotine (RTG) 4mg versus placebo (PLC) as add-on therapy with AChEI in patients with mild AD according to the consensus diagnostic criteria and MMSE score of ≥18 and ≤26 at screening. Additionally, the study aimed to test if RTG induced change in brain physiology. To this aim, we used a multimodal neurophysiological tool, such as transcranial magnetic stimulation combined with electroencephalography (TMS/EEG), to measure changes in cortical activity in the prefrontal cortex. We expected that Rotigotine could represent a valid treatment for cognitive dysfunction in AD patients, slowing the cognitive and functional decline and increasing cortical activity of the prefrontal cortex.

5.1. Primary Objective

The primary objective of this study is to test the hypothesis that therapy with Rotigotine could have a relevant clinical impact on cognitive impairment in Alzheimer’s disease patients as compared with placebo. The primary objective will be evaluated using a study endpoint at 24 weeks after initiation of treatment. The primary objective will be assessed using a Generalized Linear Mixed Model (GLMM) for repeated measures on the primary outcome: Alzheimer’s Disease Assessment Scale-Cognitive (ADAS-Cog), in which the specific hypothesis is that the cognitive decline at the end of the treatment phase for RTG will be significantly less than that for placebo.

5.2. Secondary Objective

The secondary objectives of this study are as follows:

- To test the hypothesis that RTG will slow the rate of decline associated with AD in the activities of daily living, as assessed with the Alzheimer's disease Cooperative Study - Activities of Daily Living (ADCS-ADL), using a GLMM for repeated measures.
- To assess benefit of treatment with RTG on frontal cognitive functions, as demonstrated through the Frontal assessment battery (FAB), using a GLMM for repeated measures.
- To evaluate efficacy of treatment with RTG on behavior, as demonstrated through the Neuropsychiatric Inventory (NPI), using a GLMM for repeated measures.
- To provide evidence that RTG modulates the frontal cortical activity, as evaluated in terms of cortical excitability and oscillatory activity evoked by single-pulse TMS during EEG, using a repeated-measures ANOVA. These biomarkers will be collected only in subsets of patients.

SECTION 6: INVESTIGATIONAL PLAN

6.1. Summary of Study Design

This is a 24-week, randomized, double-blind, placebo-controlled, Phase 2 study, comparing Rotigotine (Neupro, UCB pharma) with placebo for 24 weeks in approximately 100 patients with mild AD, defined as having a Visit 1 MMSE score of 18 to 26. Patients will be adaptively randomized (Lin et al. 2016) in a 1:1 ratio stratified by sex, age, APOE genotype, education and screening severity (MMSE) to one of the following treatment groups: Placebo group; RTG 4mg group. All patients started the treatment with a package containing 7 patches of RTG 2mg/placebo, and continued treatment with 6 packages, each one containing 30 patches of RTG 4mg/placebo depending on the arm of randomization. The primary hypothesis being tested is that Rotigotine will slow the cognitive and functional decline of AD as compared with placebo in patients with mild AD.

6.2. Timing of outcomes assessment

Preliminary screening

- Review of inclusion and exclusion criteria
- Informed consent obtained
- Demography
- Family and medical history
- Prior and concomitant medications
- Randomization and group assignment

Baseline evaluation (Day 1)

- Clinical assessment
- Neurophysiological assessment

Post-treatment evaluation (Day 168-week 24)

- Clinical assessment
- Neurophysiological assessment

6.3. Screening Phase

At or before Visit 1, the study will be explained to the patient and caregiver. In the screening phase, all patients will undergo an extensive clinical investigation, including interviews on their medical history, a full neurological examination, the MMSE, a complete blood screening, neuropsychological assessment, neuropsychiatric evaluation, and magnetic resonance imaging. In the 30 days before entering the study, subjects will have to be clear of treatment with any drug inducing modulatory effects on the cerebral cortex excitability, such as antidepressants, benzodiazepines, anti-epileptic drugs, or neuroleptics. All AD patients will have to show a moderate level of dementia, as assessed by a neuropsychological evaluation including the MMSE (ranging from 18 to 26) and a standardized neuropsychological battery (Carlesimo et al,1996). All the patients will perform lumbar puncture for CSF biomarkers analysis for diagnostic purposes (Dubois et al., 2014). CSF t-tau and p-tau phosphorylated at Thr181 concentrations will be determined using a sandwich ELISA (InnotesthTAU-Ag, Innogenetics, Gent, Belgium) (Brem et al., 2013). Aβ1-42

levels will be determined using a sandwich ELISA (Innotest® ß- amyloid (1–42), Innogenetics, Gent, Belgium), specifically constructed to measure Aβ containing both the first and 42nd amino acid, as previously described (Martorana et al., 2012). Patients will be excluded if they have either two or more hyperintense lesions with a diameter ≥ 10mm or more than eight hyperintense lesions with a diameter between 5 and 9 mm on dual-echo MR images (Bozzali et al, 2011). Additionally, patients with TMS-related exclusion criteria will be not included in the study: presence of pacemakers, aneurysm clips, artificial heart valves, ear implants, or foreign metal objects in the eyes, skin or body, history of seizures (Rossi et al., 2009). All participants will sign a written informed consent after receiving an extensive disclosure of the experimental details.

6.4. Evaluation Phase

During the week before starting RTG/PLC administration, and during the week at the end of 24-week treatment, AD patients will undergo clinical assessment and neurophysiological assessment in two different days.

1) Clinical Assessment: before and after the 24 weeks of treatment the ADAS-Cog, ADCS-ADL, FAB and NPI will be administered.

2) Neurophysiological assessment: for EEG-TMS recordings, a TMS-compatible EEG equipment will be used for recording EEG activity from the scalp (BrainAmp 32MRplus, BrainProducts). The EEG will be continuously acquired from 32 scalp sites positioned according to the 10-20 International System. TMS-EEG recordings will be performed over the left prefrontal cortex (PFC) and left parietal cortex (l-PPC). To precisely position the coil over the cortical sites across different sessions, a neuronavigation system (Softaxic, E.M.S.) will be used. During the TMS-EEG session, each cortical site will be stimulated with 80 single TMS pulses (ISI: 0.3 Hz) (Koch et al., 2018).

6.5. Treatment Phase

After recruitment and baseline assessments, AD patients will be assigned to RTG or PLC as add on to current AChEI therapy (donepezil or rivastigmine). We will use the Neupro® (Rotigotine Transdermal System), a transdermal delivery system that provides rotigotine, a non-ergolinic DA

agonist, currently used for treatment of Parkinson's disease. When applied to intact skin, Neupro is designed to continuously deliver rotigotine over a 24-hour period. The precise mechanism of action of rotigotine, as a treatment for Parkinson's disease, is unknown although it is suggested to be related to its ability to stimulate D2 receptors within the brain. All of the non-ergoline agonists currently in clinical use are able to bind and activate the D2-like family of dopamine receptors, although they differ in their relative efficacy at these receptors. In preclinical studies, rotigotine has been already shown to act as a strong dopamine D2 receptor agonist (Van der Weide *et al*., 1987; Tan, 2003). Moreover, in radioligand binding studies (Scheller *et al*., 2009), rotigotine exhibited high affinity for the dopamine D3 receptor (Ki 0.71 nM), D2, D5 and D4 receptors (Ki 4–15 nM) and low affinity for the dopamine D1 receptor (Ki 83 nM). Furthermore, the continuous and uniform release of rotigotine from the transdermal delivery system has been demonstrated to give stable plasma drug levels over 24 h, providing a relatively continuous, more physiological, stimulation of dopamine receptors, with potential greater benefits on disease symptoms.

All treatments will be administered for 24 weeks with no interruptions. All treatments will be administered for 24 weeks with no interruptions. Rotigotine will be administered through a 4 mg transdermal patch (Neupro, UCB pharma), after having started with a 2 mg patch for 1 week. Transdermal patches of rotigotine have a release surface area of 10 or 20 cm^2 and contain 4.5 or 9 mg of rotigotine to release respectively 2 or 4 mg during a 24-hour period when applied to intact skin. The placebo transdermal patch contained in cardboard packaging will be identical to the rotigotine except for the absence of rotigotine.

SECTION 7: STUDY POPULATION

Eligible patients will be males and females with mild to moderate AD, as specified in the entry criteria that follow. Entered patients who meet all of the inclusion criteria and are not excluded by any of the exclusion criteria will be randomized and proceed to Evaluation Phase.

7.1. Inclusion Criteria

Patients are eligible to be included in the study only if they meet all of the following criteria:

1. The patient (or if applicable the legally acceptable representative if different from the responsible caregiver) and the responsible caregiver have signed the Informed Consent Form.
2. The patient has probable AD, diagnosed according to National Institute of Neurological and Communicative Disorders and Stroke and the Alzheimer's Disease and Related Disorders Association (NINCDS-ADRDA) criteria.
3. The patient is a man or woman, aged >50 ≤ 85 years.
4. The patient has a Clinical Dementia Rating (CDR) total score of 0.5 or 1 (mild) and MMSE score of 18-26 (inclusive) at Screening.
5. Has at least one identified adult caregiver who is able to provide meaningful assessment of changes in subject behavior and function over time and provide information on safety and tolerability, and is able to verify daily compliance with study drug
6. The patient has been treated with acetylcholinesterase inhibitor (AChEI), i.e., donepezil, galantamine, or rivastigmine, at the time of screening:
   - for at least 6 months,
   - the current dosage regimen and must have remained stable for ≥ 8 weeks,
   - dosage regimen must have remained stable throughout participation in the study.
7. The patients have performed lumbar puncture for CSF biomarkers analysis for diagnostic purposes.

7.2. Exclusion Criteria

Patients will be excluded from study enrollment if they meet any of the following criteria:

1. Significant neurodegenerative disorder of the central nervous system other than Alzheimer's disease, e.g., Lewy body dementia, Parkinson's disease, multiple sclerosis, progressive supranuclear palsy, hydrocephalus, Huntington's disease, any condition directly or indirectly caused by Transmissible Spongiform Encephalopathy (TSE), Creutzfeldt-Jakob Disease (CJD), variant Creutzfeldt-Jakob Disease (vCJD), or new variant Creutzfeldt-Jakob Disease (nvCJD)

2. The patient has history of seizure (with the exception of febrile seizures in childhood)

3. Diagnostic and Statistical Manual of Mental Disorders, Fourth Edition - Text Revision (DSM IV-TR) criteria met for any of the following within specified period:

- Major depressive disorder (current)
- Schizophrenia (lifetime)
- Other psychotic disorders, bipolar disorder, or substance (including alcohol) related disorders (within the past 5 years)

4. Metal implants in the head (except dental), pacemaker, cochlear implants, or any other non-removable items that are contraindications to MR imaging.

5. Evidence of clinically significant disease including but not limited to pulmonary, gastrointestinal, renal, hepatic, endocrine, cardiovascular or metabolic disorder (Patients with controlled diabetes, or hypertension, or complete/partial right bundle branch block may be included in the study).

6. Treatment currently or within 6 months before baseline with any of the following medications:

- Typical and atypical antipsychotics (i.e. Clozapine, Olanzapine)
- monoamine oxidase B inhibitors, anticholinergics etc)
- Carbamazepine, Primidone, Pregabalin, Gabapentin
- Memantine

7.3. Screening Procedures

Eligibility of participants in the study will be based on the results of sequential screening procedures including medical history, physical examination, and neurological evaluation including MRI or PET. The screening period allows patients adequate time to decide whether they wish to participate in the study.

SECTION 8: TREATMENT

8.1. Treatments administrated

This study involves a comparison of Rotigotine treatment administered as transdermal patch for 24 weeks compared with placebo. All patients started the treatment with a package containing 7 patches of RTG 2mg/placebo, and continued treatment with 6 packages, each one containing 30 patches of RTG 4mg/placebo depending on the arm of randomization.

- Subjects will be adaptively randomized (Lin et al. 2016) in a 1:1 ratio stratified by sex, age, APOE genotype, education and screening severity (MMSE) to one of the following treatment groups: Placebo group; RTG 4mg group.
- The investigational treatment packaging has a 1-part label. A unique randomization number is printed on each label. Investigator staff identifies the investigational treatment package(s) to dispense to the patient. Labels include the following information: the name and address of the Sponsor, the study code, a unique identifier, appropriate contact information, and expiry date.
- Treatment is given to all patients for 24 weeks with no interruption.
- The investigational drug Neupro (rotigotine) is provided as transdermal patch contained in cardboard packaging. Placebo patches is identical in appearance and will be contained in identical packages.
- At each post-Baseline visit during the treatment period, the number of patches dispensed to the patient/caregiver is recorded. Patient compliance with prescribed study drug is also assessed at

each visit by questioning the patient and caregiver. Compliance data, including dates of any dose deviations and/or interruptions, and any other pertinent information are recorded in the source documentation.

- Use of the following treatments are not allowed in combination with study treatment, due to confounding of efficacy and/or potential interaction with study treatment:

  - Any investigational drug: disallowed <60gg from screening
  - General anesthetics are disallowed during the study except in case of emergency procedures requiring anesthesia. Episodic use of local anesthetics is allowed.
  - Antidepressant: stable treatment with no modification of dose with selective serotonin reuptake inhibitors (SSRIs), venlafaxine, moclobemide and mirtazapine for at least 6 months prior to the Screening Visit acceptable. Paroxetine and duloxetine should be used with caution. Dose modifications and initiation of treatment not allowed during the study.
  - Antiparkinsonian agents (e.g., levodopa, dopamine agonists, COMT inhibitors, amantadine, monoamine oxidase B inhibitors, anticholinergics etc.): disallowed for 6 months prior to screening and during the study.
  - Antipsychotics typical and atypical: disallowed for 3 months prior to screening and during the study.
  - Sedative/hypnotics: disallowed for 3 months prior to screening and during the study. Melatonin will be allowed anytime.

- The investigators or designees keep a record of all study drug received, and of all study drug dispensed to and returned by patients/caregivers.

8.2. Rationale for Selection of Doses

Neupro (Rotigotine Transdermal System) is indicated for the treatment of the signs and symptoms of idiopathic Parkinson's disease. The effectiveness of Neupro was demonstrated in randomized, controlled trials in patients with early-stage Parkinson’s disease who were not receiving

concomitant levodopa therapy as well as in patients with advanced-stage Parkinson's disease on concomitant levodopa. We chose a low 4 mg dosage because such a drug has been previously found to be effective in modulating cholinergic activity and cortical plasticity in AD patients with no relevant side effects (Martorana et al, 2013; Koch et al., 2014). We chose to test the effects of RTG, which has an impact only on a subset of dopamine receptors, and not those of levo-dopa, which has a more global DA activation, because RTG can be administered with transdermal patches and it is well tolerated in AD patients (Martorana et al, 2013; Koch et al., 2014). PLC will be administered using a transdermal patch similar to those medicated. In all cases, each daily patch will be maintained for 24 h.

SECTION 9: EFFICACY AND SAFETY EVALUATIONS: OUTCOME MEASURES

9.1. Primary Efficacy Measure

1) Global cognition [Time Frame: change from baseline to Week 24]: Alzheimer's Disease Assessment Scale - Cognitive Subscale (ADAS-Cog). The ADAS-Cog is the cognitive subscale of the ADAS (Rosen et al., 1984; Fioravanti et al., 1994) which consists of 11 tasks that measure memory, orientation, language, and praxis. The total score range is 0-70, with higher scores indicating greater impairment. ADAS-Cog scores have been shown to deteriorate by approximately 8-9 points per annum in patients with AD (Stern et al., 1994). The ADAS-Cog has a good inter-rater reliability and has been used widely as primary measure of cognitive change in contemporary studies of AD.

9.2. Secondary Efficacy Measures

1) Frontal cognitive functions [Time Frame: change from baseline to Week 24]: Frontal assessment battery (FAB). The Frontal Assessment Battery is a brief screening tool for the assessment of frontal lobe function (Appollonio et al., 2005). It is used to measure abstraction,

fluency, impulsivity and primitive reflexes in patients who have a suspected frontal lobe deficit. A higher score indicates better performance. The FAB has been used to assess the effects of rivastigmine on frontal functions in AD patients (Park et al., 2017)

2) Activities of daily living [Time Frame: change from baseline to Week 24]: Alzheimer's disease Cooperative Study - Activities of Daily Living (ADCS-ADL). The ADCS-ADL includes 23 items that were derived from a larger set of items describing performance of activities of daily living (ADL) by Alzheimer's disease patients (Galasko et al., 1997). The ADCS contained 23 items covering physical and mental functioning and independence in self-care. For each basic ADL (eating, walking, toileting, bathing, grooming, selecting, clothes), there is a forced choice of best response. All other ADL consist of a main question followed by subquestions (descriptors). The scores range from 0 to 78, with lower values indicating greater disability.

3) Neuropsychiatric evaluation [Time Frame: change from baseline to Week 24]: Neuropsychiatric Inventory (NPI). The NPI assesses behavioral disturbances in dementia. The NPI is reliable and valid (Cummings, 1994) and has been reported to be sensitive to the effects of tacrine (Kaufer et al., 1996). The NPI measures the following behavioral areas: delusions, hallucinations, agitation, depression, anxiety, euphoria, apathy, disinhibition, irritability, aberrant motor behavior, night time behaviors, and eating disorders. Symptoms are rated in terms of frequency (0-4) and severity (0-3). Total score range is 0-144, where higher scores indicate higher behavioral psychopathology.

4) Neurophysiological markers of frontal lobe cortical activity [Time Frame: change from baseline to Week 24]: TMS-evoked cortical potentials assessed over the prefrontal cortex (PFC). The combined use of transcranial magnetic stimulation (TMS) during electroencephalography (EEG) allows to measure the cortical activity of the prefrontal cortex in AD patients (Kähkönen et al., 2005; Julkunen et al., 2008). We employ TMS-evoked cortical responses (i.e., TEPs) as a novel probe of dopamine-agonist induced cortical excitability changes. As biomarkers we will measure neurophysiological changes induced by dopamine-agonist over the left dorsolateral prefrontal

cortex (PFC), and over the left posterior parietal cortex (l-PPC), by evaluating the cortical excitability and oscillatory activity evoked by single-pulse TMS during EEG recordings. We chose to stimulate the left DLPFC since we want to assess the spatial specificity of our treatment, whereas the left PPC is chosen as a control site. Two sets of outcome measures will be obtained assessing cortical excitability (global mean field power-GMFP) and cortical oscillatory activity.

9.3. Safety Evaluations

Investigators are responsible for monitoring the safety of patients who have entered this study. The investigator is responsible for the appropriate medical care of patients during the study. The investigator remains responsible for following, through an appropriate health care option, AEs that are serious or that caused the patient to discontinue before completing the study. The patient should be followed until the event is resolved or explained. Frequency of follow-up evaluation is left to the discretion of the investigator.

9.4. Adverse Events and Other Safety Measures

A clinical study AE is any untoward medical event associated with the use of a drug or drug delivery system in humans. AE collection begins after the patient has signed informed consent and has received study drug. If a patient experiences a AE after signing informed consent, but before receiving study drug, the event will not be collected unless the investigator feels the event may have been caused by a protocol procedure.

At each clinic visit, AEs will be recorded, vital signs will be measured and physical and neurological examination will be performed. An independent Data Monitoring Committee will monitor the patients' safety according to the Data Monitoring Committee Charter.

SECTION 10. INFORMED CONSENT, ETHICAL REVIEW AND REGULATORY CONSIDERATIONS

11.1. Informed Consent

The principal investigator is responsible for ensuring that the patient understands the potential risks and benefits of participating in the study, including answering any questions the patient may have throughout the study and sharing in a timely manner any new information that may be relevant to the patient's willingness to continue his or her participation in the trial.

The Informed Consent Form (ICF) will be used to explain the potential risks and benefits of study participation to the patient/caregivers in simple terms before the patient is entered into the study, and

to document that the patient is satisfied with his or her understanding of the risks and benefits of participating in the study and desires to participate in the study. The investigator is responsible for ensuring that informed consent is given by each patient or legal representative. This includes obtaining the appropriate signatures and dates on the ICF prior to the performance of any protocol procedures and prior to the administration of investigational product.

11.2. Ethical Review and Regulatory Considerations

This study will be conducted in accordance with:

1) consensus ethics principles derived from international ethics guidelines, including the Declaration of Helsinki and Council for International Organizations of Medical Sciences (CIOMS) International Ethical Guidelines

2) Institutional Review Board (IRB) Guidelines

3) Applicable laws and regulations.

The investigator or designee will promptly submit the protocol to applicable ERB. An identification code assigned by the investigator to each patient will be used in lieu of the patient's name to protect the patient's identity when reporting AEs and/or other trial-related data.

11.3. Investigator Information

Physicians with expertise in neurology or geriatrics who have clearly documented extensive experience in AD trials will participate as investigators in this clinical study. In addition, licensed clinicians (neuropsychologists) who have clearly documented extensive experience in AD trials

may participate as investigators in this clinical study. Cognitive assessments must be administered by an individual trained in the use of these instruments. Neurophysiological recordings and analysis must be performed by experts in this field. All lumbar punctures must be performed by an appropriately trained individual.

2. STATISTICAL ANALYSIS PLAN

2.1. Determination of Sample Size

A total of 94 randomly assigned patients (47 per group) were planned on the basis of our previous study in which we assessed the effects of rotigotine on cortical plasticity and cognitive functions in a small sample of Alzheimer's disease patients (Koch et al., 2014). In that pilot study, a significant (although unpowered) difference was observed in pre-post (12 weeks) treatment with rotigotine in n=7 patients in both the cognitive measures MMSE (mean pre=21.73, SD=4.14; mean post=23.23, SD=3.93) and in FAB (mean pre=10.85, SD=4.43; mean post=12.32, SD=4.48). Treatment duration of the present study is twice larger than the one of the pilot study thus a larger effect size is expected. However, for precautionary reasons, we consider the same effect sizes found in the previous work (equal to 0.48 for MMSE and equal to 0.42 for FAB computed as post-pre means over standard deviation of differnce post-pre), for the sample size calculation of the present study. In detail, adopting a two-tailed paired t-test, with type I error alpha=0.05 and a plausible correlation between pre-post measured variables of 0.7, the FAB effect size equal to 0.42 requires a minimum sample of n=46 for reaching a power of 0.8. For MMSE, this sample size allows to reach a power of 0.9. The minimum total sample size was then augmented up to N=92 considering the matched placebo group.

2.2. Method of Assignment to Treatment

Subjects who meet all criteria for enrollment will be randomized to double-blind treatment. Randomization will be performed and assigned independently by a statistician working in an independent institution, held centrally, and not divulged to any other person involved in the trial until after database lock. In order to obtain homogeneous and balance study groups in terms of age, sex and APOE carriers, an adaptive randomization will be adopted; more specifically, a covariate-adaptive randomization will be performed, i.e. a randomization procedure that uses past group assignments and subject covariate values to select the probability of future group assignments, with the objective to balance group assignments within covariate profiles (Lin et al. 2016). The simple

randomization procedure could lead to unbalanced groups (with respect to specific key patients features) when the sample size is relatively small: the adaptive procedure allowed to overcome this drawback through a pseudo-random assignment that took into account the key features (age [55-70 vs >70], sex [male vs female] and APOE [carriers vs non-carriers]) of the subjects previously assigned into the study groups.

2.3 Statistical and Analytical Plans

2.3.1. General Considerations

Unless otherwise noted, all tests of treatment effects will be conducted at a 2-sided alpha level of 0.05; 2-sided confidence intervals will be displayed with a 95% confidence level. All tests of interactions between treatment and other factors will be conducted at an alpha level of 0.05. All analyses will follow the intent-to-treat (ITT) principle unless otherwise specified. An ITT analysis is an analysis of data by the groups to which subjects are assigned by random allocation, even if the subject does not take the assigned treatment, does not receive the correct treatment, or otherwise does not follow the protocol.

2.3.2. Analysis Populations

Baseline characteristics as well as the co-primary and secondary efficacy measures will be summarized for the ITT and per-protocol populations by treatment group and overall. Tabulations of the number and percentage of subjects included in each analysis set, by treatment group and overall, will be provided. Reasons for exclusion from analysis datasets will also be provided.

2.3.3. Patient Characteristics

The patient’s age, gender, height, body weight, tobacco use, alcohol use, caffeine use, years of education, work status, time since onset of first AD symptoms, time since diagnosis, MMSE at Visit 1, APOE4 carrier status (carrier [ε2/ε4, ε3/ε4, ε4/ε4], noncarrier [ε3/ε3, ε2/ε2]), APOE4 genotype (ε2/ε4, ε3/ε4, ε4/ε4, no ε4) and AChEI use at baseline will be recorded. Baseline characteristics will

be summarized for the ITT and per-protocol populations by treatment group and overall. Summaries will include descriptive statistics for continuous and categorical measures. Pearson's chi-square test will be used for treatment group comparisons of categorical data. For continuous data, t-test or corresponding non-parametric test (Mann-Whithney) will be used for the group comparing.

2.3.4. Analysis of Efficacy Outcomes

Normality assumption of end-points variables will be assessed by inspection of the distribution plots and by Kolmogorov-Smirnov and Shapiro-Wilk tests. Preliminary descriptive analyses will be performed by means, frequencies, standard deviations (SD) and percentages. Comparison of socio-demographic and clinical features between the study groups at baseline will be performed through t-test or Mann-Whitney tests.

The longitudinal assessment of the end-points across groups will be performed through Generalized Linear Mixed Model (GLMM) for repeated measures with random intercept and random slope to account for individual differences at baseline as well as for individual change after the treatment. GLMMs will be applied to ADAS-Cog and the other efficacy outcome measures, ADCS-ADL, FAB and NPI, as dependent variables and group, time and group x time interaction as independent factors. In detail, GLMMs for Gaussian data with identity link function will be applied for ADAS-Cog, ADCS-ADL and FAB, whereas GLMM for Poisson data, with log-link function, will be used for NPI. The GLMMs on MMSE, ADAS-Cog and FAB will be adjusted for age and education. To evaluate the treatment effects on TMS/EEG data, we will use repeated-measures ANOVAs with between-subjects factor group and within-subject factors time.

To evaluate the treatment effects on cortical activity, a repeated-measures ANOVA with between-subjects factor "group" and within-subject factors "time" will be planned. Repeated-measures mixed ANOVA with between-subjects factor "group" and within-subject factors "time" will be planned to evaluate the treatment effects on oscillatory activity. Prior to undergoing ANOVA procedures, normal distribution of clinical, behavioral and neurophysiological data will be assessed

by means of Shapiro-Wilks' test. The sphericity of the data will be tested with Mauchly's test; when sphericity is violated (i.e. Mauchly's test < 0.05) the Greenhouse–Geisser correction will be used. Pairwise comparisons will be corrected by the Bonferroni method. Safety analyses for the treatment period will include comparisons between rotigotine and placebo groups by 2-sided 0.05 significance level Chi-square tests. Statistical analyses will be performed with IBM SPSS statistics, version 24.0 and R software; significance alpha level test is set up at 0.05.

2.3.5. Adjustment for covariates

Possible confounders will be chosen based on literature. The evaluation of the effect of such confounders on primary and secondary outcomes will be carried out through an ad-hoc linear and generalized linear model and a corresponding correlation analysis of residuals.

REFERENCES

Appollonio I, Leone M, Isella V, Piamarta F, Consoli T, Villa ML, Forapani E, Russo A, Nichelli P. The Frontal Assessment Battery (FAB): normative values in an Italian population sample. Neurol Sci. 2005; 26(2):108-16.

Berlanga ML, Simpson TK, Alcantara AA. Dopamine D5 receptor localization on cholinergic neurons of the rat forebrain and diencephalon: a potential neuroanatomical substrate involved in mediating dopaminergic influences on acetylcholine release. J Comp Neurol. 2005; 492(1):34-49.

Bozzali M, Padovani A, Caltagirone C, Borroni B. Regional grey matter loss and brain disconnection across Alzheimer disease evolution. Curr Med Chem. 2011; 18(16): 2452-2458.

Carlesimo GA, Caltagirone C, Gainotti G. The Mental Deterioration Battery: normative data, diagnostic reliability and qualitative analyses of cognitive impairment. The Group for the Standardization of the Mental Deterioration Battery. Eur Neurol. 1996; 36(6): 378–384.

Cummings JL, Mega M, Gray K, Rosenberg-Thompson S, Carusi DA, Gornbein J. The Neuropsychiatric Inventory: comprehensive assessment of psychopathology in dementia. Neurology 1994; 44(12): 2308-14.

Di Lazzaro V, Oliviero A, Tonali PA, Marra C, Daniele A, Profice P, Saturno E, Pilato F, Masullo C, Rothwell JC. Noninvasive in vivo assessment of cholinergic cortical circuits in AD using transcranial magnetic stimulation. Neurology 2002; 59(3):392-7.

Dubois B, Feldman HH, Jacova C, et al. Advancing research diagnostic criteria for Alzheimer's disease: the IWG-2 criteria. Lancet Neurol 2014; 13(6):614-29.

Fioravanti M, Nacca D, Buckley AE, Ferrario E, Varetto O, Mogni P, Fabris F. The Italian version of the Alzheimer's Disease Assessment Scale (ADAS): psychometric and normative characteristics from a normal aged population. Arch Gerontol Geriatr. 1994; 19(1):21-30.

Galasko D, Bennett D, Sano M, et al. An inventory to assess activities of daily living for clinical trials in Alzheimer’s disease. Alzheimer Dis Assoc Disord 1997;11: Suppl 2: S33-S39.

Goldman-Rakic PS. The "psychic" neuron of the cerebral cortex. Ann NY AcadSci.1999; 868:13-26.

Goldman-Rakic PS, Muly EC 3rd, Williams GV. D(1) receptors in prefrontal cells and circuits. Brain Res Brain Res Rev. 2000; 31(2-3):295-301.

Goldsmith SK, Joyce JN. Dopamine D2 receptors are organized in bands in normal human temporal cortex. Neuroscience. 1996; 74(2):435-51.

Guzmán-Ramos K, Moreno-Castilla P, Castro-Cruz M, McGaugh JL, Martínez-Coria H, LaFerla FM, Bermúdez-Rattoni F. Restoration of dopamine release deficits during object recognition memory acquisition attenuates cognitive impairment in a triple transgenic mice model of Alzheimer's disease. Learn Mem. 2012;19(10):453-60.

Himeno E, Ohyagi Y, Ma L, Nakamura N, Miyoshi K, Sakae N et al. Apomorphine treatment in Alzheimer mice promoting amyloid-b degradation. Ann Neurol. 2011; 69(2): 248-256.

Itoh A, Nitta A, Nadai M, Nishimura K, Hirose M, Hasegawa T, Nabeshima T. Dysfunction of cholinergic and dopaminergic neuronal systems in beta-amyloid protein--infused rats. J Neurochem. 1996; 66(3):1113-7.

Joyce JN, Kaeger C, Ryoo H, Goldsmith S. Dopamine D2 receptors in the hippocampus and amygdala in Alzheimer's disease. Neurosci Lett. 1993; 14;154(1-2):171-4.

Joyce JN, Myers AJ, Gurevich E. Dopamine D2 receptor bands in normal human temporal cortex are absent in Alzheimer's disease. Brain Res. 1998a; 16;784(1-2):7-17.

Joyce JN, Murray AM, Hurtig HI, Gottlieb GL, Trojanowski JQ. Loss of dopamine D2 receptors in Alzheimer's disease with parkinsonism but not Parkinson's or Alzheimer's disease. Neuropsychopharmacology. 1998b;19(6):472-80.

Julkunen P, Jauhiainen AM, Westerén-Punnonen S, Pirinen E, Soininen H, Könönen M, Pääkkönen A, Määttä S, Karhu J. Navigated TMS combined with EEG in mild cognitive impairment and Alzheimer's disease: a pilot study. J Neurosci Methods. 2008; 30;172(2):270-6.

Kähkönen S, Komssi S, Wilenius J, Ilmoniemi RJ. Prefrontal transcranial magnetic stimulation produces intensity-dependent EEG responses in humans. Neuroimage. 2005; 15;24(4):955-60.

Kaufer DI, Cummings JL, Christine D. Effect of tacrine on behavioral symptoms in Alzheimer's disease: an open-label study. J Geriatr Psychiatry Neurol. 1996; 9(1):1-6.

Kemppainen N, Laine M, Laakso MP, Kaasinen V, Någren K, Vahlberg T, Kurki T, Rinne JO. Hippocampal dopamine D2 receptors correlate with memory functions in Alzheimer's disease. Eur J Neurosci. 2003;18(1):149-54.

Koch G, Bonnì S, Pellicciari MC, Casula EP, Mancini M, Esposito R, Ponzo V, Picazio S, Di Lorenzo F, Serra L, Motta C, Maiella M, Marra C, Cercignani M, Martorana A, Caltagirone C, Bozzali M. Transcranial magnetic stimulation of the precuneus enhances memory and neural activity in prodromal Alzheimer's disease. Neuroimage. 2018; 169:302-311.

Koch G, Di Lorenzo F, Bonnì S, Giacobbe V, Bozzali M, Caltagirone C, Martorana A. Dopaminergic modulation of cortical plasticity in Alzheimer's disease patients. Neuropsychopharmacology. 2014;39(11):2654-61.

Koch G, Di Lorenzo F, Bonnì S, Ponzo V, Caltagirone C, Martorana A. Impaired LTP- but not LTD-like cortical plasticity in Alzheimer’s disease patients. J Alzheimers Dis. 2012; 31(3): 593-599.

Kumar U, Patel SC. Immunohistochemical localization of dopamine receptor subtypes (D1R-D5R) in Alzheimer's disease brain. Brain Res. 2007; 1131(1):187-96.

Lin J, Lin LA, Sankoh S. A general overview of adaptive randomization design for clinical trials. J Biom Biostat 2016; 7(2):294.

Martorana A, Mori F, Esposito Z, Kusayanagi H, Monteleone F, Codecà C et al. Dopamine modulates cholinergic cortical excitability in Alzheimer’s disease patients. Neuropsychopharmacology, 2009; 34 (10): 2323-2328.

Martorana A, Esposito Z, Koch G. Beyond the cholinergic hypothesis: do current drugs work in Alzheimer's disease? CNS Neurosci Ther. 2010; 16(4):235-45.

Martorana A, Esposito Z, Di Lorenzo F, Giacobbe V, Sancesario GM, Bucchi G, Bonnì S, Bernardini S, Sorge R, Sancesario G, Bernardi G, Caltagirone C, Koch G. Cerebrospinal fluid levels of Aβ42 relationship with cholinergic cortical activity in Alzheimer's disease patients. J Neural Transm. 2012; 119(7):771-8.

Martorana A, Di Lorenzo F, Esposito Z, Lo Giudice T, Bernardi G, Caltagirone C et al. Dopamine D2-agonist rotigotine effects on cortical excitability and central cholinergic transmission in Alzheimer's disease patients. Neuropharmacology, 2013; 64: 108-113.

Park KW, Kim EJ, Han HJ, et al. Efficacy and tolerability of rivastigmine patch therapy in patients with mild-to-moderate Alzheimer's dementia associated with minimal and moderate ischemic white matter hyperintensities: A multicenter prospective open-label clinical trial. PLoS One. 2017;12(8):e0182123.

Park JH, Myung W, Choi J, et al. Extrapyramidal Signs and Cognitive Subdomains in Alzheimer Disease. Am J Geriatr Psychiatry. 2016; 24(7):566-74.

Paspalas CD1, Goldman-Rakic PS. Presynaptic D1 dopamine receptors in primate prefrontal cortex: target-specific expression in the glutamatergic synapse. J Neurosci. 2005; 2;25(5):1260-7.

Pizzolato G, Chierichetti F, Fabbri M, Cagnin A, Dam M, Ferlin G, Battistin L. Reduced striatal dopamine receptors in Alzheimer's disease: single photon emission tomography study with the D2 tracer [123I]-IBZM. Neurology. 1996; 47(4):1065-8.

Rosen WG, Mohs RC, Davis KL. A new rating scale for Alzheimer's disease. Am J Psychiatry. 1984; 141(11):1356-64.

Rossi S, Hallett M, Rossini PM, Pascual-Leone A; Safety of TMS Consensus Group. Safety, ethical considerations, and application guidelines for the use of transcranial magnetic stimulation in clinical practice and research. Clin Neurophysiol. 2009;120(12):2008-2039.

Ryoo HL, Joyce JN. Loss of dopamine D2 receptors varies along the rostrocaudal axis of the hippocampal complex in Alzheimer's disease. J Comp Neurol. 1994; 1;348(1):94-110.

Scheller D, Ullmer C, Berkels R, Gwarek M, Lübbert H. The in vitro receptor profile of rotigotine: a new agent for the treatment of Parkinson's disease. Naunyn Schmiedebergs Arch Pharmacol. 2009;379(1):73-86.

Stern RG, Mohs RC, Davidson M, Schmeidler J, Silverman J, Kramer-Ginsberg E, Searcey T, Bierer L, Davis KL. A longitudinal study of Alzheimer's disease: measurement, rate, and predictors of cognitive deterioration. Am J Psychiatry. 1994;151(3):390-6.

Tan EK. Dopamine agonists and their role in Parkinson's disease treatment. Expert Rev Neurother. 2003; 3(6):805-10.

Tanaka Y, Meguro K, Yamaguchi S, Ishii H, Watanuki S, Funaki Y et al. Decreased striatal D2 receptor density associated with severe behavioral abnormality in Alzheimer's disease. Ann Nucl Med. 2003; 17(7):567-573.

Van der Weide J, De Vries JB, Tepper PG, Horn AS. In vitro binding of the very potent and selective D-2 dopamine agonist, [3H]N-0437 to calf caudate membranes. Eur J Pharmacol. 1987; 134(2):211-9.

Zhang J, Xiong B, Zhen X, Zhang A. Dopamine D1 receptor ligands: where are we now and where are we going. Med Res Rev. 2009; 29(2):272-94.
