# Supplementary material for: Effect of Rotigotine vs Placebo on Cognitive Functions Among Patients With Mild to Moderate Alzheimer Disease: A Randomized Clinical Trial
Source: JAMA Netw Open. 2020 Jul 15;3(7):e2010372. doi: 10.1001/jamanetworkopen.2020.10372 (PMC7364345; doi:10.1001/jamanetworkopen.2020.10372)
Supplement: Supplement 2. — eAppendix 1. Supplementary Statistics of Clinical Data eAppendix 2. Supplementary Statistics of TMS-EEG Data eFigure 1. Box Plot of Clinical Variables eFigure 2. Global Mean Field Power and Oscillatory Activity Evoked From the Left Posterior Parietal Cortex in the Rotigotine and Placebo Groups Before and After Completion of the Trial eTable 1. Association Between Age, Education and Secondary End Points Evaluated by Linear Regression Model for MMSE, ADAS, FAB, ADCS-ADL and Poisson Regression for NPI eTable 2. Generalized Linear Mixed Model With Poisson Distribution for NPI Subscales eMethods. Supplementary Methods for TMS-EEG Recordings and Processing eReferences. [file jamanetwopen-3-e2010372-s002.pdf]

Supplementary Online Content

Koch G, Motta C, Bonnì S, et al. Effect of rotigotine vs placebo on cognitive functions among patients with mild to moderate Alzheimer disease: a randomized clinical trial. *JAMA Netw Open*. 2020;3(7):e2010372. doi:10.1001/jamanetworkopen.2020.10372

eAppendix 1. Supplementary Statistics of Clinical Data

eAppendix 2. Supplementary Statistics of TMS-EEG Data

eFigure 1. Box Plot of Clinical Variables

eFigure 2. Global Mean Field Power and Oscillatory Activity Evoked From the Left Posterior Parietal Cortex in the Rotigotine and Placebo Groups Before and After Completion of the Trial

eTable 1. Association Between Age, Education and Secondary End Points Evaluated by Linear Regression Model for MMSE, ADAS, FAB, ADCS-ADL and Poisson Regression for NPI

eTable 2. Generalized Linear Mixed Model With Poisson Distribution for NPI Subscales

eMethods. Supplementary Methods for TMS-EEG Recordings and Processing

eReferences.

This supplementary material has been provided by the authors to give readers additional information about their work.

© 2020 Koch G et al. *JAMA Network Open.*

eAppendix 1. Supplementary Statistics of Clinical Data

Distribution features, Gaussianity assumptions and association with socio-demographic (age and education) variables for the primary and secondary end-point. All clinical variables (except NPI) have low variability: coefficients of variation CV = (SD/mean) * 100 were equal to 13,37,27,21 and 82 for MMSE, ADAS-Cog, FAB, ADCS-ADL, and NPI respectively. Moreover, all the distributions appear symmetric and without outliers (that are present only in NPI distribution which is notably a skewness discrete count distribution and have to be modeled accordingly by Poisson distribution).

© 2020 Koch G et al. *JAMA Network Open.*

eAppendix 2. Supplementary Statistics of TMS-EEG Data

ANOVA performed on mean GMFP amplitude, evaluated over left dorsolateral prefrontal cortex, revealed a significant treatment x time interaction [F(1,38)=11.235; p=0.002], due to a significant increase of cortical activity observed after 24 weeks of RTG treatment. Post-hoc analysis showed a significant difference between pre and post rotigotine treatment (p=0.001) and between post rotigotine and post placebo treatment (p=0.006). No significant difference was observed between baseline of each treatment and between pre and post placebo treatment. Additionally, ANOVA performed on global evoked oscillatory responses (EOR) values revealed a significant treatment x time interaction [F (1,38) =6.837; p=0.013], due to an enhancement of cortical oscillatory activity in all frequency bands after 24 weeks of rotigotine treatment. Post-hoc analysis showed a general increase between pre and post rotigotine treatment (p=0.062) paralleled by a decrease between pre and post placebo treatment (p=0.084), in all frequency bands. No effect was observed between the baselines of the two treatments, i.e. pre rotigotine and pre placebo (p>0.05). Finally, no significant cortical changes were found when evaluating l-PPC (see supplementary Figure S2). These results indicated that the rotigotine treatment was able to induce topographically-specific effects on cortical activity and oscillatory activity at frontal level.

© 2020 Koch G et al. *JAMA Network Open.*

eFigure 1. Box Plot of Clinical Variables

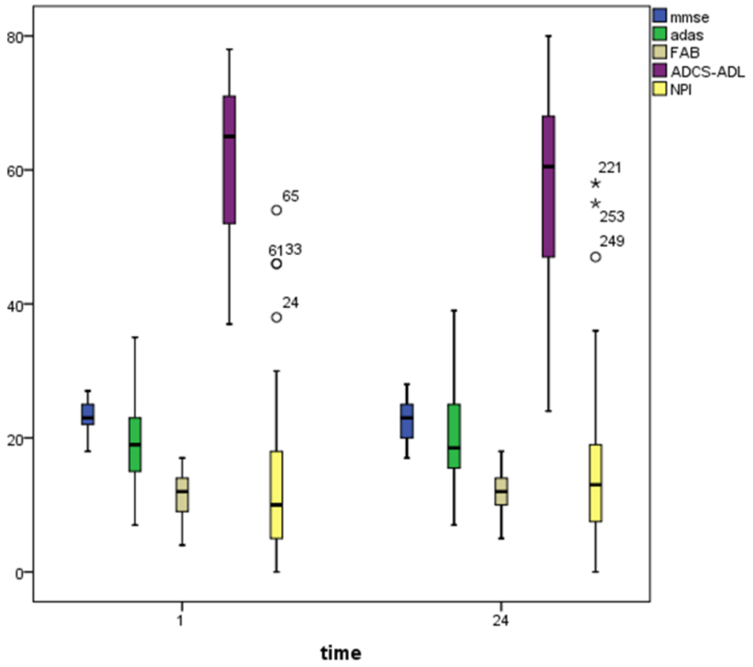

Legend to Supplementary Figure S1

Box-plots of the clinical variables at baseline and at 24 weeks and a description of the coefficient of variation CV, symmetry and outliers of the MMSE, ADAS-Cog, FAB, ADCS-ADL, and NPI variables.

© 2020 Koch G et al. *JAMA Network Open.*

eFigure 2. Global Mean Field Power and Oscillatory Activity Evoked From the Left Posterior Parietal Cortex in the Rotigotine and Placebo Groups Before and After Completion of the Trial

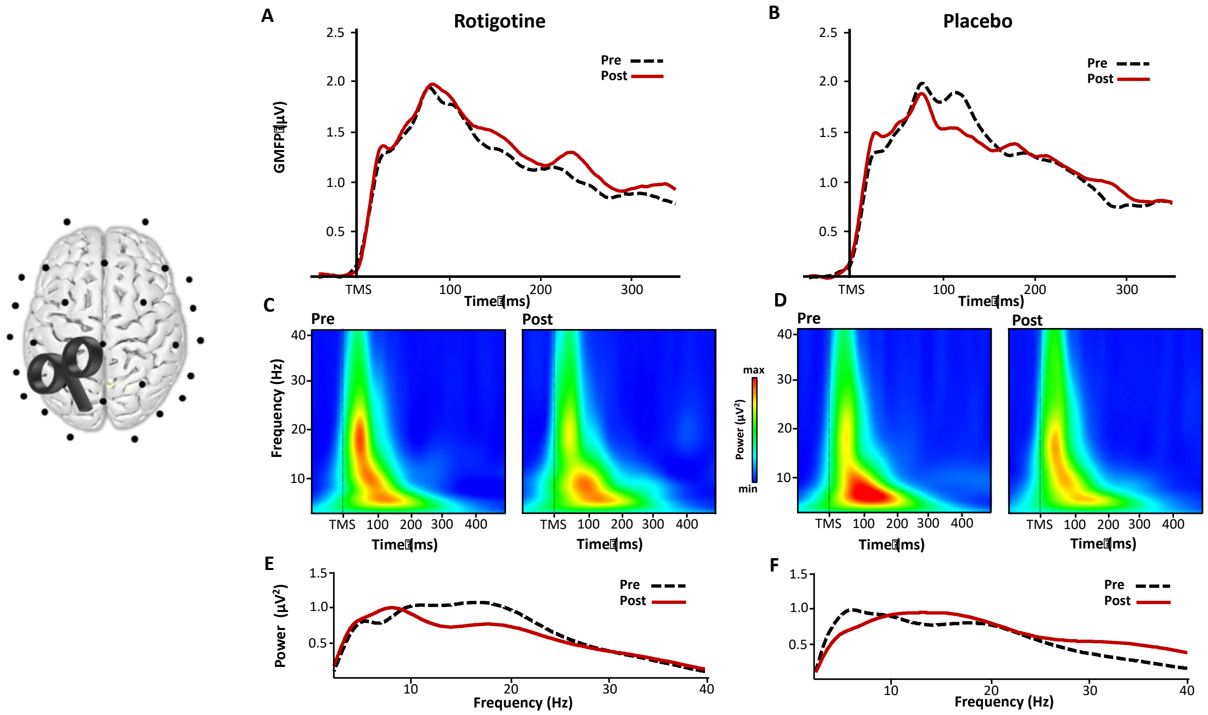

Legend to Supplementary Figure S2

Global mean field power (upper panels A-B) and oscillatory activity (middle and lower panels C-F) evoked from the left posterior parietal cortex in the rotigotine and placebo groups before and after completion of the trial. In the upper panel, black dashed lines depict the activity evoked before and red lines after the 24-week period of treatment respectively in rotigotine group (panel A) and placebo group (panel B). The middle panels show oscillatory activity in the group of patients treated with rotigotine (panel C) and with placebo (panel D). The lower panels show the power spectrum profile of evoked oscillatory activity depicted in middle panels. In the panel E, black line depicts the activity evoked before and red line after the 24-week period of treatment with rotigotine, whereas in the panel F, black dashed line depicts the activity evoked before and blue line after the 24-week period of treatment with placebo.

© 2020 Koch G et al. *JAMA Network Open.*

eTable 1. Association Between Age, Education and Secondary End Points Evaluated by Linear Regression Model for MMSE, ADAS, FAB, ADCS-ADL and Poisson Regression for NPI

	AGE		EDUCATION		GAUSSIANITY TESTS ON RESIDUALS	
	**Std beta**	**pvalue**	**Std beta**	**pvalue**	**K-S test**	**S-W test**
**MMSE**	0.183	**0.014**	0.278	**<0.001**	0.200	0.383
**ADAS-Cog**	-0.155	**0.044**	-0.126	**0.099**	0.175	0.076
**FAB**	0.211	**0.005**	0.260	**0.001**	0.200	0.242
**ADCS-ADL**	-0.037	0.629	0.078	0.316	0.048	0.095
**NPI**	-0.004	0.222	-0.005	0.299	0.002#	0.004#

Std beta: standardized beta coefficient; K-S_ Kolmogorov-Smirnov test; S-W: Shapiro-Wilk test
NPI is a count variable and residual are expected to be non-gaussian distributed.

Age and education are significantly associated with MMSE, ADAS-Cog and FAB, whereas no association was found for ADCS-ADL and NPI. After adjustment for age and education, MMSE, ADAS-Cog and FAB have Gaussian distributed residuals thus models with Gaussian distribution was adopted for further analyses on these outcomes. Same model, although on the borderline, was applied also to ADCS-ADL without adjustment for age and education. Finally, generalized (Poisson regression) linear models, without adjustment for age and education, was applied for NPI.

© 2020 Koch G et al. *JAMA Network Open.*

eTable 2. Generalized Linear Mixed Model With Poisson Distribution for NPI Subscales

	**Group [Rotigotine vs placebo]**		**TIME**		**Group X Time**	
	**F statistic (dof)**	**pvalue**	**F statistic (dof)**	**pvalue**	**F statistic (dof)**	**pvalue**
Delusions	2.101 (1,144)	0.149	2.101 (1,144)	0.005	2.383 (1,144)	0.402
Hallucinations	0.140 (1,184)	0.122	0.140 (1,184)	0.203	0.707 (1,184)	0.653
Agitation_aggression	0.020 (1,143)	0.381	0.020 (1,143)	0.366	0.203 (1,143)	0.148
Depression_disphoria	0.07 (1,144)	0.578	0.07 (1,144)	0.511	0.586 (1,144)	0.445
Anxiety	0.010 (1,144)	0.241	0.010 (1,144)	0.691	0.810 (1,144)	0.370
Elation_euphoria	8.146 (1,184)	0.005	2.503 (1,184)	0.115	3.931 (1,184)	**0.049**
Apathy_indifference	1.814 (1,184)	0.180	0.780 (1,184)	0.378	0.043 (1,184)	0.836
Disinhibition	0.047 (1,144)	0.828	3.459 (1,144)	0.065	0.022 (1,144)	0.882
Irritability_lability	1.068 (1,144)	0.303	1.945 (1,144)	0.165	0.042 (1,144)	0.838
Aberrant_motor	0.073 (1,144)	0.788	0.218 (1,144)	0.641	2.407 (1,144)	0.123
sleep_disorders	6.447 (1,144)	0.012	0.208 (1,144)	0.649	0.208 (1,144)	0.649
Appetite_eating_disorders	0.527 (1,144)	0.0469	1.649 (1,144)	0.201	2.383 (1,144)	0.125

(dof): degree of freedom

© 2020 Koch G et al. *JAMA Network Open.*

eMethods. Supplementary Methods for TMS-EEG Recordings and Processing

To assess the changes in cortical activity induced by dopaminergic agonist rotigotine, TMS was delivered in single pulses during EEG over the dorsolateral prefrontal cortex and posterior parietal cortex of the left hemisphere (DLPFC and PPC) of a subgroup of AD patients (N=20 assigned to RTG treatment and N=20 assigned to PLC treatment). We chose to stimulate the left DLPFC as a cortical target site of the dopaminergic projection, whereas the left PPC was chosen as a control site. A neuronavigation system (SofTaxic; EMS) coupled with a Polaris Vicra infrared camera was used to ensure the same cortical targets of stimulation before and after each treatment. During TMS-EEG recordings, all the patients wore in-ear plugs continuously playing a white noise, with intensity adjusted for each patient in order to mask the TMS click and avoid possible auditory event related responses (Massimini et al., 2005).

Eighty TMS single-pulses were applied, during an EEG recording with open eyes, with an intensity of 90% of the resting motor threshold, as assessed over the left motor cortex. A TMS-compatible EEG equipment (BrainAmp 32MRplus, BrainProducts GmbH, Munich, Germany) was used to record the EEG activity from 29 scalp sites positioned according to the 10-20 International System. TMS-compatible Ag/AgCl pellet electrodes were mounted on an elastic cap, while additional electrodes were used as ground and reference. The ground electrode was positioned in AFz, while the reference one was positioned on the tip of the nose. Horizontal and vertical eye movements were detected by recording the electrooculogram (EOG), to monitor participant behavior on line and to reject off-line the trials with ocular artifacts. The EEG and EOG signals were band-pass filtered at 0.1–1000 Hz and digitized at a sampling rate of 5 kHz. Skin/electrode impedance was maintained below 5 kΩ.

TMS-EEG data were analyzed off-line (Brain Vision Analyzer, Brain Products GmbH, Munich, Germany), with different approaches both in spatio/temporal domain for evaluating cortical activity changes and in time/frequency domain for evaluating cortical oscillatory changes. First, the artifact induced by the TMS pulse was removed using an interpolation for a conservative interval from 1 ms before to 10 ms after the TMS pulse. Consequently, the first 10 ms following the pulse were excluded

© 2020 Koch G et al. *JAMA Network Open.*

from the analysis. Bad channels were interpolated using spherical interpolation function when needed. To identify and remove components reflecting residual muscle activity, eye movements, blink-related activity, and residual TMS-related artifacts we used an independent component analysis (INFOMAX-ICA). After these steps, the signal was re-referenced offline to the mean signal across all electrodes, downscaling (1000 Hz), band-pass filtered (1 and 80 Hz, Butterworth zero phase filters, with a 50 Hz notch filter). Epochs with excessively noisy EEG, eye-movement artifacts or muscle artifacts were excluded from the analysis after a visual inspection.

Cortical activity changes were evaluated averaging TMS-evoked response in the whole epoch from 100 before to 500 ms after single TMS pulse. All epochs were baseline corrected to a time period of 100 ms recorded before TMS pulse. The time course of the global cortical response evoked by TMS was determined by calculating the global mean field power (GMFP) (Lehmann and Skrandies, 1980) and averaging its amplitude between 10 to 300 ms following TMS-pulse (Casula et al., 2016).

Cortical oscillatory changes were evaluated by detecting the evoked oscillatory response in epochs starting 1 s before to 1 s after the TMS pulse. A time/frequency decomposition based on a complex Morlet wavelet transform (2–40 Hz, 38 frequency steps, cycle= 3.5) was applied to averaged epochs in each patients normalizing the data to a window between 700 to 400 ms preceding TMS onset. The global evoked oscillatory response (EOR) (Pellicciari et al., 2017) was computed by averaging the oscillatory activity of all cortical channels. To minimize the effect of possible artifacts occurring at the time of stimulation, the frequency values were calculated by averaging the EOR values over a 20–250 ms time window, corresponding to the main activity evoked by single TMS pulse. Subsequently, the spectral power in the frequency ranges between 2 and 4 Hz (delta), 4–7 Hz (theta), 8–12 Hz (alpha) and 13–30 Hz (beta) was extracted from the wavelet dataset.

Statistical analysis

To assess the treatment effects on cortical activity, we performed a repeated-measures ANOVA on GMFP amplitude with a between-subjects factor treatment (RTG and PLC) and a within-subject factor time (pre- and post-treatment), separately for the two stimulation sites (l-DLPFC and l-PPC).

© 2020 Koch G et al. *JAMA Network Open.*

To assess the effects on cortical oscillatory activity, we performed a repeated-measures ANOVA on global EOR with a between-subjects factor treatment (RTG and PLC), a within-subject factors time (pre- or post-treatment) and frequency band (delta, theta, alpha and beta). Sphericity of data was tested with Mauchly's test; when sphericity was violated (i.e. Mauchly's test <0.05), the Greenhouse-Geisser correction was used. Bonferroni-adjusted pairwise comparisons were then performed. The p-values less than 0.05 were considered significant. All statistical analyses were performed using SPSS 23.0 (SPSS Inc., Chicago, IL, USA).

© 2020 Koch G et al. *JAMA Network Open.*

eReferences

Massimini M, Ferrarelli F, Huber R, Esser SK, Singh H, Tononi G. Breakdown of cortical effective connectivity during sleep. Science. 2005 Sep 30;309(5744):2228-32.

Lehmann D, Skrandies W. Reference-free identification of components of checkerboard-evoked multichannel potential fields. Electroencephalogr Clin Neurophysiol. 1980;48:609-21.

Casula EP, Pellicciari MC, Picazio S, Caltagirone C, Koch G. Spike-timing-dependent plasticity in the human dorso-lateral prefrontal cortex. Neuroimage. 2016 Dec;143:204-213.

Pellicciari MC, Veniero D, Miniussi C. Characterizing the Cortical Oscillatory Response to TMS Pulse. Front Cell Neurosci. 2017 Feb 27;11:38.

© 2020 Koch G et al. *JAMA Network Open.*
