# Supplementary material for: Effect of Rotigotine vs Placebo on Cognitive Functions Among Patients With Mild to Moderate Alzheimer Disease: A Randomized Clinical Trial
Source: JAMA Netw Open. 2020 Jul 15;3(7):e2010372. doi: 10.1001/jamanetworkopen.2020.10372 (PMC7364345; doi:10.1001/jamanetworkopen.2020.10372)
Supplement: Supplement 3. — Data Sharing Statement [file jamanetwopen-3-e2010372-s003.pdf]

Data Sharing Statement

Koch. Effect of Rotigotine vs Placebo on Cognitive Functions Among Patients With Mild to Moderate Alzheimer Disease. *JAMA Netw Open*. Published July 15, 2020. 10.1001/jamanetworkopen.2020.10372

Data
Data available: Yes
Data types: Deidentified participant data
How to access data: G.KOCH@HSANTALUCIA.IT
When available: With publication

Supporting Documents
Document types: None

Additional Information
Who can access the data: esearchers whose proposed use of the data has been approved
Types of analyses: for any purpose
Mechanisms of data availability: with a signed data access agreement
